# Antioxidant effect of yeast on lipid oxidation in salami sausage

**DOI:** 10.3389/fmicb.2022.1113848

**Published:** 2023-01-16

**Authors:** Yingli Liu, Yating Cao, Kalekristos Yohannes Woldemariam, Shengjie Zhong, Qinglin Yu, Jing Wang

**Affiliations:** ^1^China-Canada Joint Lab of Food Nutrition and Health (Beijing), Key Laboratory of Special Food Supervision Technology for State Market Regulation, Beijing Engineering and Technology Research Center of Food Additives, Beijing Technology and Business University, Beijing, China; ^2^Delisi Group Co., Ltd., Weifang, China

**Keywords:** yeast, lipid oxidation, salami, volatile compounds, antioxidant potential

## Abstract

Salami is a kind of fermented meat product with rich nutrition and unique flavor. Because it is rich in fat, it is easy to oxidize to produce bad flavor. Compared with the way of adding artificial or natural antioxidants to reduce the degree of sausage oxidation, the antioxidant characteristics of developing the starter itself deserve more attention. In this study, firstly the antioxidant activities of 5 strains of yeast were measured *in vitro*, and then the mixture of yeast and Lactobacillus rhamnosus YL-1 was applied to fermented sausage model. The effect of the starter in the sausage model was investigated through physicochemical parameters, degree of fat oxidation, free fatty acid content, and though volatile flavor compound analysis, sensory evaluation and various indexes after storage were observed. Metagenomics was used to explore metabolic pathways, functional genes and key enzymes related to lipid oxidizing substances in sausage in yeast. The results showed that Wickerhamomyces anomalus Y12-3 and Y12-4 had strong tolerance to H2O2, and had higher SOD and CAT enzyme activities. The addition of yeast effectively reduced the material value of peroxidation value and active thiobarbiturate in salami. In flavor analysis, the content of flavor compounds associated with lipid oxidation, such as hexanal, heptanal, nonanal and (E)-2-decenal were significantly lower with the use of Debaryomyces hansenii Y4-1 and Y12-3. Meanwhile, the possible pathways of yeast metabolism of flavor substances related to lipid oxidation (mainly aldehydes) were discussed with the help of metagenomic techniques. According to the results of metagenomics, fatty acid degradation (ko00071) metabolic pathway was related to the degradation of aldehydes through aldehyde dehydrogenase, which was the potential key enzyme.

## 1. Introduction

Fermented sausage refers to the meat products with long shelf life, unique flavor, texture, and color made by mixing raw meat with starter, seasoning and spices under specific temperature and humidity conditions and pouring them into casings (Huang and Huan, 2016). The important components in fermented meat products (protein and fat) are easily oxidized and degraded in the process of production and processing, thus affecting the quality of products. In the process of fermentation and maturation, proteins were hydrolyzed into short peptides and free amino acids, which are degraded into aldehydes, acids and esters under the action of microorganisms. Moderate degradation of protein can improve the nutritional value and flavor of the product, while excessive oxidation of protein will adversely affect the texture, water retention and flavor of meat, and oxidative induction of protein may also affect the digestibility and reduce the nutritional value of meat products (Berardo et al., 2015). During fermentation process besides the protein hydrolysis the lipid oxidation hydrolysis also plays an important role in the production of flavor compounds. In addition to directly producing volatile flavor compounds such as hexanal, 2-nonenal, 2, 4-nonadienal, ethyl butyrate and 1-octene-3-ol, lipid oxidation degradation products can further participate in the Maillard reaction, thus giving fermented meat products unique and rich flavor (Liu et al., 2021b). Moderate fat oxidation can improve the meat flavor, quality, but excessive oxidation results in rancidity, loss of color and texture, and shorten the shelf life. It also affects consumer acceptance and highly contribute to the production of toxic substances such as malondialdehyde, amyl aldehyde, 4-hydroxy nonyl aldehyde which are a big concern to human health (Cao et al., 2019). Therefore, in processing and circulation of fermented meat products, keeping the balance of protein and fat hydrolysis and oxidation is a big concern.

The application of synthetic antioxidants is widely used technique, while it has its own drawbacks as mainly it is in general synthetic and are mainly a concern related to the cause of cancer. Among some of these antioxidants are butylated hydroxytoluene (BHT), butylated hydroxyanisole (BHA), and tert-butylhydroquinone (TBHQ) are mainly a concern to cancer while the use of ascorbic acid is considered to be the safest (Seo et al., 2021). With the decrease in synthetic antioxidants application, the screening and application of starter cultures for fermented sausage production with antioxidant capacity are attracting more and more attention. Zhang et al. (2017) measured the antioxidant activity of lactic acid bacteria isolated from fermented sour meat, among which, *L. curvatus* SR6 had high 2-diphenyl-1-picrylhydrazyl (DPPH) free radical scavenging and reducing ability, and *L. paracasei* SR10-1 had high hydroxyl free radical scavenging activity and lipid peroxidation inhibition ability. Coppola et al. (1997) isolated 138 strains of *Staphylococcus* and *Micrococcus* from molisana, a traditional Italian fermented sausage, all of which had Catalase activity test (CAT) activity and moreover could reduce nitrate to nitrite in most strains. Perea-Sanz et al. (2020) found that sausages inoculated with *D. Hansenii* maintained a lower degree of thiobarbituric acid reactive substances (TBARS), while inhibiting nitrite oxidation and promoting the formation of flavor substances such as 3-methylbutanal in sausages. This makes *D. Hansenii* as one of the potential strains that can be applied as a starter culture in fermented sausage products. Even though it has a big potential, the study on the antioxidant activity is very limited.

Although some strains such as Lactobacillus (Zhang et al., 2017), Staphylococcus (Coppola et al., 1997), Aspergillus (Arora and Chandra, 2010), Yeast (Banwo et al., 2021), and other microorganisms also show some antioxidant potential, while there is still lack of systematic and in-depth research in the antioxidant activity of starters applicable in fermented sausage production. Based on the understanding of this point, the antioxidant activities of five yeast strains were measured in vitro first. Considering the lack of acid producing and bacteriostatic effects of lactic acid bacteria, if yeast is used alone for sausage production, it will lead to quality and safety problems in the sausage. Therefore, lactic acid bacteria and yeast were mixed together to apply to fermented salami sausage. At the same time, lactic acid bacteria used alone is set as the experimental control to obtain more accurate results. The yeast antioxidant activity, degree of lipid oxidation and volatile flavor components of sausage after fermentation and storage were detected. The possible pathway of yeast metabolism of fatty acid through aldehydes degradation was analyzed by metagenomic technology.

## 2. Materials and methods

### 2.1. Yeast strains and culture media

Six species of starter cultures, designated *Debaryomyces hansenii* Y3-1, *Debaryomyces hansenii* Y4-1, *Wickerhamomyces anomalus* Y12-2, *Wickerhamomyces anomalus* Y12-3, *Wickerhamomyces anomalus* Y12-4, and *Lactobacillus rhamnosus* YL-1 were previously isolated from traditional fermented foods (Liu et al., 2015). They were stored at the School of Food and Health, Beijing Technology and Business University (BTBU), Beijing, China. *L. rhamnosus* YL-1 has been proved to have strong acid production capacity; in addition, it has been shown to contribute to good flavor formation. YL-1 were stored in glycerin and Man-Rogosa-Sharpe (MRS) broth medium mixture at −80°C until use, whereas yeasts were stored at −80°C on the glycerin and yeast-peptone-dextrose (YPD) broth medium mixture until use.

### 2.2. Determination of tolerance of hydrogen peroxide

After activation, the yeast was inoculated with the same OD value (OD600 = 1.0) into the sterilized YPD liquid medium which added H_2_O_2_ solution (the final concentration of H_2_O_2_ was 0.0, 2.0, 4.0, 6.0, or 8.0 mmol/L), and cultured at 28°C and 150 rpm for 24 h. The absorbance A_*x*_ of culture medium was measured at wavelength 600 nm (Yang et al., 2012). The group without H_2_O_2_ solution was used as blank control with the absorbance labeled as A_0_. The same volume of liquid medium YPD was used as blank-zero with absorbance labeled as A_*j*_. The survival rate of yeast was calculated according to the following formula.


(1) $$\mathrm{Survival}\,\mathrm{Fraction}\,\left(\mathrm{SF}\right)=\frac{\text{Ax}-A\,\text{j}}{\text{A}\,0-A\,\text{j}}\times 100\%$$

### 2.3. Determination of antioxidant activity of yeast *in vitro*

After yeast strains were activated twice on YPD plate, single colonies were selected and inoculated into 50 mL YPD liquid medium, and cultured overnight at 28°C and 150 rpm. Then, the fermentation broth was transferred to 50 mL YPD liquid medium with the same OD value (OD_600_ = 1.0), and cultured at 28°C and 150 rpm for 72 h.

After the end of fermentation, the fermentation liquid was centrifuged (4°C, 20700 *× g*, 5 min). The precipitation was washed with sterile deionized water and the yeast concentration was adjusted to 10^9^ CFU/mL. At the same time, a part of cell suspension was taken for ultrasonic crushing for 40 min (650 W, 30 s on, 30 s off) under ice bath condition. After crushing, the mixture was centrifuged (4°C, 20700 *× g*, 5 min), and the supernatant was taken as the sample of intracellular cell-free extracts (Chen et al., 2015). The above two samples were used for the determination of subsequent antioxidant experiments.

### 2.4. Free radical scavenging capacity

The hydroxyl free radical scavenging rate and superoxide anion free radical scavenging rate of each component of yeast fermentation for 72 h were determined by referring to the method of hydroxyl free radical determination kit and superoxide anion free radical determination kit (Nanjing Jiancheng Technology Co., Ltd., Nanjing, China).

### 2.5. Total antioxidant capacity and antioxidant enzyme activity

The total antioxidant capacity and antioxidant enzyme activity was conducted using the manual for T-AOC kit and Glutathione peroxidase (GPX) kit which were purchased from Nanjing Jiancheng Technology Co., Ltd. The catalase activity test was performed using Catalase (CAT) kit and the superoxide dismutase activity was conducted following Superoxide dismutase (SOD) kit purchased from Beijing Solarbio Technology Co., Ltd.

### 2.6. Sausage manufacture

The sausage was divided into following seven batches: batch CK (without inoculation), batch LGG inoculated with about 10^7^ CFU/g of *L. rhamnosus* YL-1, batch S1 inoculated with about 10^7^ CFU/g of *L. rhamnosus* YL-1 and 10^6^ CFU/g of *D. hansenii* Y3-1, batch S2 inoculated with about 10^7^ CFU/g of *L. rhamnosus* YL-1 and 10^6^ CFU/g of *D. hansenii* Y4-1, batch S3 inoculated with about 10^7^ CFU/g of *L. rhamnosus* YL-1 and 10^6^ CFU/g of *W. anomalus* Y12-2, batch S4 inoculated with about 10^7^ CFU/g of *L. rhamnosus* YL-1 and 10^6^ CFU/g of *W. anomalus* Y12-3, batch S5 inoculated with about 10^7^ CFU/g of *L. rhamnosus* YL-1 and 10^6^ CFU/g of *W. anomalus* Y12-4. The formulation of the sausages was modified based on the formulation of Huang et al. (2020), Liu et al. (2021a). In detail it includes 80% lean pork meat, 20% pork back fat, 3% NaCl, 0.3% sucrose, 0.3% black pepper, 0.3% white pepper, 0.2% glucose, 0.1% garlic powder, 0.05% D-sodium erythorbate, and 0.015% sodium nitrite. Lean meat, fat, ingredients, and starter cultures were mixed with a blender at 4°C. The mixture was then stuffed into a collagen casing (200 g of meat-mixture for each sausage), and placed in a fermentation chamber.

All sausages were fermented for 20 h at 23°C with 80% relative humidity (RH) and for 24 h at 21°C with 65% RH. Then the sausages are ripened for 24 h at 20°C with 67% RH, 24 h at 19°C with 69% RH, 24 h at 18°C with 71% RH, 24 h at 16°C with 73% RH, 24 h at 15°C with 74% RH, 24 h at 14°C with 76% RH, 24 h at 12°C with 77% RH, and kept at 10°C with 73% RH until the end of 23 days. The samples in storage were kept sealed in the dark at room temperature until 60 days, others sausage was vacuum packaged and frozen at -80°C for subsequent analyses. At each corresponding fermentation time (0, 5, 10, 16, 23, and 60 days), three randomly selected sausages of each treatment were used to analyze pH, a_*w*_, POV, TBARS, free fatty acids measurement, volatile compound analysis and sensory analysis.

### 2.7. Determination of physical and chemical properties of sausage

According to the method of Beck et al. (2021), the pH, and water activity a_*w*_ were measured during sausage ripening using a pH meter (Testo 205, AG, Testo, Lenzkirch, Germany) and an Aqualab 4TE water activity meter (Decagon Devices Inc., Pullman, WA, United States), respectively.

### 2.8. Determination of the peroxide value

Peroxide values for sausages were determined using the method of Vareltzis et al. (2008) with a few modifications. Take an appropriate amount. A broken sausage samples 2.5 g was transferred into a 50 mL centrifuge tube and, add 3 times of petroleum ether with respect to the sample weight, mixed well and left for extraction for 12 h. The sample solution is filtered by a funnel containing anhydrous sodium sulfate, and the filtrate is evaporated under reduced pressure at 39°C, and the residue is the sample to be tested.

The 1 mL of samples to be tested and 50 μL ferrous chloride (3.5 g/L) solution were transferred to centrifugal tube, then the volume was adjusted to 10 mL by mixture of dichloromethane:methanol (v :v = 7:3). Add 50 μL 30% potassium thiocyanate solution, mixed well and the mixture was allowed to stand for 5 min at room temperature. The absorbance of the supernatant was measured at wavelength 500 nm, mixture of dichloromethane:methanol (v :v = 7:3) was used to zero the instrument. POV value was calculated by standard curve, and the result was expressed as meq/kg sample.


(2) $$\text{POV}=\frac{\text{C}}{\text{m}\times 111.68}$$

Where, C is the mass of iron obtained against the standard curve, expressed in μg, m is the mass of the sample which represented by 1 mL of samples to be tested, expressed in g, and 116.68 is the conversion factor.

### 2.9. Determination of thiobarbituric acid reactive substances

The TBARS was conducted following the method of Filho et al. (2021) with some modifications. In detail adding 5.00 g of broken sausage sample into 100 mL conical flask containing 50 mL of 75.00 g/L trichloroacetic acid solution (including 1.00 g/L ethylenediaminetetraacetic acid disodium). The mixture sealed and shake well then incubated in a thermostatic oscillator at 50°C for 30 min. The mixture is filtrated when it cools to room temperature and 5 mL of filtrate was transferred into a tube containing 5 mL of 2.88 g/L thiobarbituric acid (TBA) solution. The sample solution mixed, sealed, allowed to react in a 90°C-water bath for 30 min. After reaction take the tube out, and allowed to cool to room temperature. The absorbance of the cooled reaction solution was measured at 532 nm. The content of TBARS was determined by comparing the standard curve of malondialdehyde, which was expressed as mg of malondialdehyde/1.00 kg sausage sample (mg/kg).

### 2.10. Determination of free fatty acid

By referring to Feng et al. (2015) with some modifications, the lipids and free fatty acids were extracted and separated. In detail, 5.0 g of broken sausage samples were placed in 100 mL beaker, then add 60 mL of chloroform/methanol solution (v:v = 2:1). After mixing, the samples were homogenized (6000 rpm, 20 s) and broken twice in ice bath. The homogenates was made to stand in a fume hood for 1 h and then filtered. Filtrate was collected and 0.2-fold volume of 0.9% normal saline was added. The mixture was centrifuged for 10 min at 3000 *× g*, 4°C. The underlying organic phase was collected and evaporated into oil droplets in a vacuum at 44°C to serve as a lipid extraction sample.

Free fatty acids were separated as follows. The 100 mg lipids were weighed in a 2 mL centrifuge tube and dissolved using 1 mL chloroform and vortexed for mixing. The mixture was transferred to an aminopropyl-silica gel cartridges (SPE columns, Kangyuan Techbio Biological Technology Co., Ltd.) which activated with 1 mL chloroform. After activation, the free fatty acids in the SPE column were eluted with 3 mL 2 % acetic acid-diethyl ether solution.

The separated free fatty acid solution was dried by blowing dry nitrogen. The dried sample is mixed with 20 μL 2, 2-dimethoxy-propane (to absorb trace water produced in the process of methyl esterification) and 2 mL 14 % boron trifluoride-methanol solution, mixed and reacted in water bath at 60°C for 1 h. After methyl esterification and cooling, 1 mL n-heptane and 1 mL ultrapure water were added and dissolved using vortex. After standing for stratification, the upper organic phase was collected and dried with a nitrogen blower. Then the final volume was made up to 0.5 mL by n-heptane, then detected using GC-MS (Gas-chromatography-mass-spectrometry) detection.

The free fatty acids were separated and identified by GC-MS (8890 GC System, 5977B MSD) equipped with a DB-WAX capillary column (30 m × 250 μm × 0.25 μm, Agilent Technologies, Santa Clara, CA, United States). The GC conditions were as follows: Helium (purity of 99.99%) was used as a carrier gas at a constant flow rate of 1.0 mL/min. The inlet temperature was set to 250°C with a solvent delay of 2 min, kept at 1 μL for injection volume, and the split ratio was 5:1. The column initial temperature was 100°C, increased to 200°C at a rate of 10°C/min for 5 min, then further increased to 220°C at a rate of 1°C/min for 5 min (Huang et al., 2013).

The MS conditions were as follows. The auxiliary heater temperature and ion source temperature were respectively set to 240°C and 230°C, and the four-stage rod temperature was 150°C. MS fragmentation was detected in electron-impact (EI) mode (ionization energy of 70 eV) with an acquisition range from 40 to 500 m/z in full-scan mode. The experimental results were qualitatively analyzed by Supelco 37 Component FAME Mix (Supelco, Bellefonte, PA, United States; catalog No. 47885-U) and NIST 14 library, and quantitatively analyzed by peak area method.

### 2.11. Determination of volatile compounds

The Solid phase micro-extraction (SPME) method was used to extract and quantitate the aroma compounds according to the method of Li et al. (2015). Briefly, 3.0 g of minced sausage was weighed precisely and placed in a 20 ml headspace vial. Immediately after, 1 μL of internal standard (o-Dichlorobenzene, 1.306 μg/μL in methanol) injected quickly, The vial was placed in an HH-series digital constant-temperature water bath and incubated at 60°C for 30 min. Then volatile compounds were extracted with a extraction head (DVB/CAR/PDMS, 50/30 μm) for 30 min at 60°C.

Volatile compounds in sausage were separated and identified by GC-MS (8890 GC System, 5977B MSD) equipped with a InertCap WAX capillary column (60 m × 0.25 mm × 0.25 μm; GL Sciences, Tokyo, Japan). The GC conditions were as follows: Helium (purity of 99.999%) was used as a carrier gas at a constant flow rate of 1.6 mL/min. The inlet temperature was set to 250°C with desorbed 5 min. The column initial temperature was kept at 40°C for 3 min, increased to 180°C at a rate of 3°C/min for 3 min, then to 230°C at a rate of 10°C/min for 5 min. The ion source temperature was set to 230°C. MS fragmentation was detected in electron-impact (EI) mode (ionization energy of 70 eV) with an acquisition range from 45 to 500 m/z in full-scan mode (Martín et al., 2003). The retention time was calculated and compared with NIST 14 library to identify the peaks, and the ratio of peak area to internal standard peak area was calculated to determine the quantity.

### 2.12. Sensory analysis

Sensory evaluation of seven groups of sausage samples was carried out after fermentation. Sensory assessment was performed by 39 trained team members. Sausage samples are sliced into 2 mm slices and placed on white plates. Samples were randomly labeled with three digits, and each sample was evaluated three times. Cleanse the palate between samples was performed by ingesting salt-free biscuits and water. Odor (cheese, fruity, rancid, oily), texture, taste (sour, salty, mellow), and overall acceptability of the samples were evaluated according to a 7-point scale from 1 to 7, in which odor and taste indicators were scored according to the intensity of perception from low to high, and liking index was scored according to the degree of liking (Liu et al., 2018).

### 2.13. Metagenomic sequencing

The S4 group (Y12-3) and S5 group (Y12-4) of fermented sausages were selected for metagenomic sequencing in this experiment. After they were matured (23 days), they were crushed by a crusher and stored at −80°C for standby. Metagenomic sequencing was performed by the Shanghai Majorbio Bio-Pharm Technology Co., Ltd.

### 2.14. Statistical analysis

Three replicates were set for each treatment in this experiment. SPSS 25.0 (SPSS Inc., Chicago, IL, United States) were used for statistical significance analysis of experimental data, and GraphPad Prism (Prism v8.0, GraphPad) was used for drawing.

## 3. Results and discussion

### 3.1. Comparison of tolerance of different yeast to H_2_O_2_

As an important eukaryote, yeast is faced with various pressures such as oxidative stress during its growth. H_2_O_2_ is a strong oxidant, which can be converted into hydroxyl radical under the catalysis of metal ions, and because of its toxicity to proteins, lipids, DNA, RNA, and other molecules, so the resistance of yeast to oxidative stress (such as H_2_O_2_) can reflect its antioxidant properties.

**FIGURE 1:**
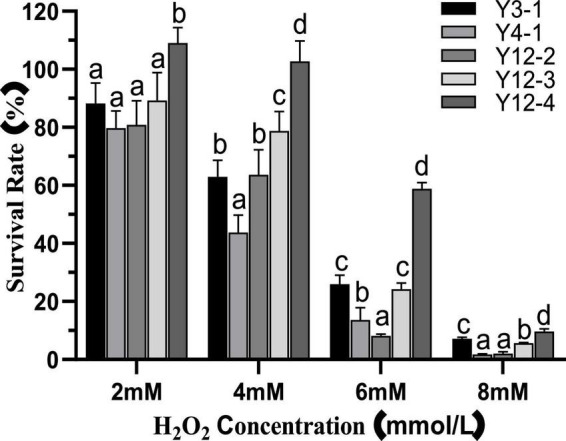
Survival rate of yeast at different concentrations of H_2_O_2_. Different letters (a–d) indicated that there was significant difference in survival rate between different yeast groups at the same H_2_O_2_ concentration (*p* < 0.05).

The effects of different concentrations of H_2_O_2_ on the survival rate of yeast strains were shown in Figure 1. Compared with the SF (100%) of the control group, the Y12-4 increased to a certain extent after 24 h treatment with 2.0 mM H_2_O_2_, which may be due to CAT in the yeast cells decomposed H_2_O_2_ into O_2_, thereby promoting its growth. This conclusion is similar to that of Wang et al. (2020). In contrast, the SF of the other four yeast strains was inhibited at 2.0 mM H_2_O_2_. Growth of all yeasts was inhibited when the concentration of H_2_O_2_ reached 6 mM. Among them, Y12-4 has the highest SF (58.89%), followed by Y3-1 (25.94%) and Y12-3 (24.29%). When the concentration of H_2_O_2_ was 8 mM, the SF of Y12-4 remained 9.64%. In conclusion, Y12-4 was the most tolerant to H_2_O_2_, followed by Y3-1 and Y12-3. While, strains Y4-1 and Y12-2 were the most sensitive to H_2_O_2_.

### 3.2. Comparison of free radical scavenging ability of different yeasts

**FIGURE 2:**
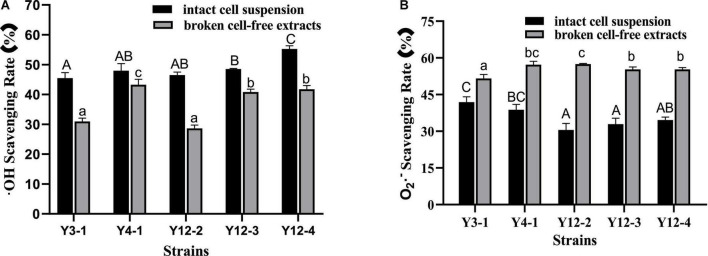
Free radical scavenging rate of different yeasts. **(A)** ^•^ OH Scavenging Rate; **(B)** O_2_^•^^–^ Scavenging Rate. Different capital letters indicated that there was significant difference in free radical scavenging rate among different intact cell suspension (*p* < 0.05). Different lowercase letters indicated that there was significant difference in the free radical scavenging rate among different broken cell-free extracts (*p* < 0.05).

Hydroxyl free radicals are an extremely active reactive oxygen species with strong oxidation capacity, which can easily cause damage to macromolecules of biological cells, thus affecting the normal function of cells. The hydroxyl radical scavenging ability of the intact cell suspension and the broken cell-free extracts of 5 yeast strains was measured, and the results were shown in Figure 2A. The hydroxyl radical scavenging capacity of 5 yeast strains was significantly different (*p* < 0.05). The hydroxyl radical scavenging rate of the intact cell suspension exceeded 45%, while that in the broken cell-free extracts was between 28 and 44 %. Among them, the intact cell suspension scavenging rate of Y12-4 was the highest, reaching 55.27%, and the scavenging rate in the broken cell-free extracts of Y4-1 was the highest, reaching 43.23%. Overall, the hydroxyl radical scavenging rates of Y3-1 and Y12-2 were significantly lower than that of other strains (*p* < 0.05).

Superoxide anion radical (O_2_^–^) is a relatively weak oxidant. Although it cannot directly induce the oxidation of lipids, it can undergo Fenton reaction in the presence of metal ions, forming strongly oxidized hydroxyl radicals and then participate in oxidation. The same way with the hydroxyl free radical scavenging activity the superoxide anion radical scavenging ability was measured for five yeast strains with whole cell and broken cell as shown in Figure 2B. The scavenging ability of superoxide anion radical of 5 yeast strains was significantly different (*p* < 0.05). In terms of different components, contrary to the results of hydroxyl radical scavenging ability, the scavenging rate of broken cell-free extracts exceeded 51%, which was stronger than that of intact cell suspension. The scavenging rate of Y12-2 broken supernatant was the highest, reaching 57.48%. The scavenging rate of superoxide anion free radicals in intact bacterial suspension was 30−42%, and the scavenging rate of Y3-1 intact bacterial suspension was 41.90%, which was significantly higher than that of other strains (*p* < 0.05).

In conclusion, there were significant differences in the free radical scavenging activities of intact cell suspension and broken cell-free extracts of different yeasts. The free radical scavenging activity of intact cell suspension may be related to its antioxidant substances (such as enzymes, polyphenols, and other yeast secretions) and polysaccharide proteins on the cell wall surface (Jaehrig et al., 2008). Li et al. (2012) found that Polysaccharides or proteins on the surface of intact cells of plantarum C88 participate in the DPPH scavenging effect. After removing these compounds, the DPPH scavenging ability of suspension is significantly reduced. The free radical scavenging activity of the broken supernatant may be attributed to the role of GSH-Px, SOD, CAT and other antioxidant enzymes. In general, the hydroxyl free radical scavenging ability of intact cell suspension is higher than that of the corresponding broken cell-free extracts, which may be due to the thick cell wall of yeast, and makes it difficult for ultrasonic crushing to release all the antioxidant substances stored in the cells into the supernatant, so that the free radical scavenging activity of the broken cell-free extracts is lower.

### 3.3. Comparison of total antioxidant capacity of different yeasts

**FIGURE 3:**
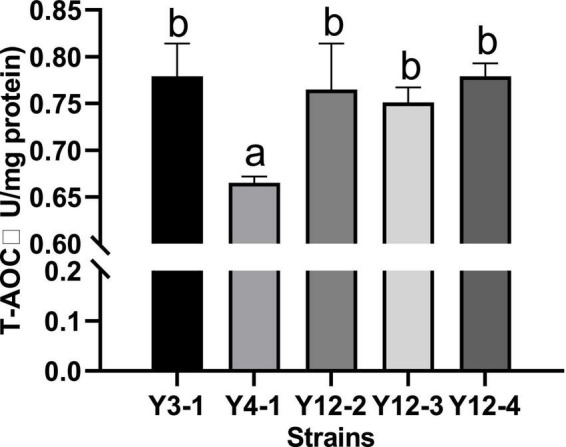
Determination of total antioxidant capacity (T–AOC) of different yeasts. Different letters indicated significant difference in total antioxidant capacity among different strains (*p* < 0.05).

The total antioxidant capacity (T-AOC) is the result of the combined action of antioxidant enzymes and non-enzymatic antioxidant substances in the tested samples, as well as the ability to scavenge free radicals, chelate metal ions and decompose peroxide. The T-AOC of the broken cell-free extracts of 5 yeast strains was measured, and the results were shown in Figure 3. All the broken cell-free extracts of different yeast had a certain total antioxidant capacity, indicating that there were substances with antioxidant activity in the supernatant after crushing. However, except Y4-1, which had a low total antioxidant activity (0.779 U/mg protein), the total antioxidant capacity of other strains higher and had no significant difference (*p* < 0.05).

### 3.4. Comparison of main antioxidant enzyme activities of different yeasts

Antioxidant enzymes, mainly including CAT, SOD, and GPX, play an important role in scavenging free radicals. The higher the enzyme activity, the stronger the antioxidant capacity. The activity results of main antioxidant enzymes in the broken cell-free extracts of these 5 yeast strains are shown in Table 1.

**TABLE 1:** The main antioxidant enzyme activities in the broken cell-free extracts (*U/mg protein*).

	Y3-1	Y4-1	Y12-2	Y12-3	Y12-4
CAT	91.70 ± 3.05ab	93.09 ± 0.28b	88.31 ± 1.10a	92.12 ± 0.99b	100.44 ± 2.08c
SOD	7.36 ± 0.28c	5.13 ± 0.54b	3.85 ± 0.62a	6.75 ± 0.25c	10.04 ± 0.24 days
GPX	n.d.	n.d.	Trace	n.d.	n.d.

Different lowercase letters indicated that there was significant difference in antioxidant enzyme activity among different strains (*p* < 0.05). n.d., indicates no detection.

Catalase activity test can catalyze the decomposition of H_2_O_2_ into H_2_O and O_2_. In this study, the CAT enzyme activity of these 5 strains ranged from 88.31 to 100.44 U/mg protein of which Y12-4 had the highest enzyme activity, reaching 100.44 ± 2.08 U/mg protein (*p* < 0.05), which was consistent with its H_2_O_2_ tolerance. Superoxides in almost all cells and cellular organisms are eliminated by SOD. In this study, the SOD activity of these 5 strains ranged from 3.85 to 10.04 U/mg protein, and Y12-4 had the highest SOD activity, reaching 10.04 ± 0.24 U/mg protein (*p* < 0.05). GPX is an important hydroxyl radical scavenger. GPX activity wasn’t detected in all yeast strains except Y12-2, which may be due to the sensitivity of the detection method is low or the GPX activity is too low to be detected.

Each yeast strain has its own advantages and disadvantages in different *in vitro* antioxidant test indexes, and there is a lager differences between the *in vitro* antioxidant test conditions and the actual application environment of fermented sausage. For example, its inhibition or promotion of lipid and protein oxidation in fermented sausage is also related to lipase and protease activities and metabolic regulation. Therefore, all the 5 yeast strains were applied to the production of fermented sausage to further explore their effects on lipid oxidation, free fatty acid changes and volatile flavor substances.

### 3.5. Analysis of pH value of fermented sausage in each group

LAB produces a large amount of lactic acid through carbohydrate metabolism at initial stage of sausage fermentation, rapidly reducing the pH value of sausage, thus inhibiting the growth of spoilage organisms and promoting the formation of flavor and color of sausage.

**FIGURE 4:**
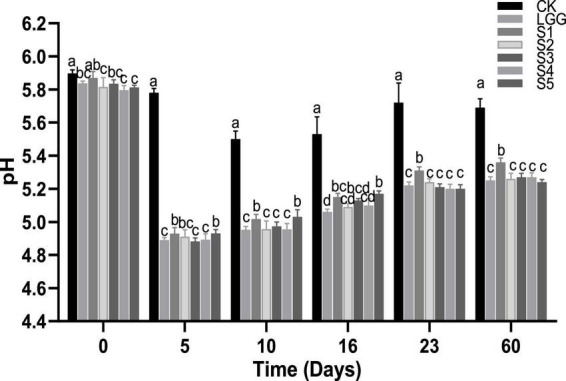
Evolution of pH value during the ripening of the different samples of sausage. Different letters indicate that there is significant difference in pH value between different groups of fermented sausage at the same time (*p* < 0.05).

As shown in Figure 4, the pH value of ground meat on 0 days was about 5.8. On the 5th day of fermentation, the pH value of the sausage group with *L. rhamnosus* YL-1 was decreased to about 4.9, which was significantly lower than that of the blank group CK (*p* < 0.05), indicating that YL-1 had good acid-producing capacity in the sausage. When the sausages were matured after 23 days of fermentation, there was no significant difference (*p* < 0.05) in pH value of fermented sausage groups except for CK group and S1 group, which was around 5.6 and 5.2, respectively. The increase of pH value in S1 group may be related to the metabolism of lactic acid by *D. hansenii* Y3-1 (He et al., 2017). On the whole, the pH value of fermented sausage in each group showed a process of first decreasing and then slowly increasing (after 10 days of fermentation). This may be due to the degradation of the protein in fermented sausage by microorganisms or endogenous protease to produce basic amino acids, biological amines and TVB-N, resulting in a slow increase in pH value (Liu et al., 2013).

### 3.6. Analysis of a_*w*_ value of fermented sausage in each group

**FIGURE 5:**
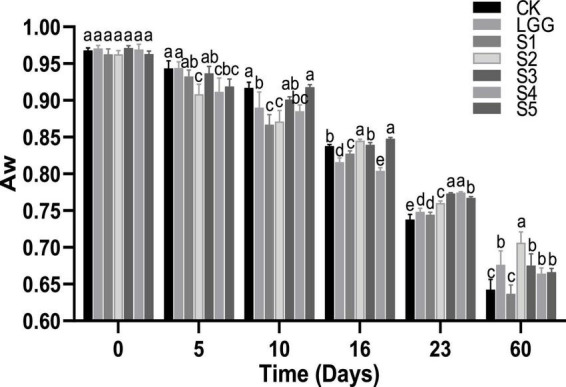
Evolution of a_w_ value during the ripening of the different samples of sausage. Different letters indicate that a_w_ value of fermented sausage in different groups has significant difference at the same time (*p* < 0.05).

The a_*w*_ directly affects the growth and metabolism of microorganisms. It is generally believed that when a_*w*_ is lower than 0.90, it can effectively inhibit the growth of spoilage and pathogenic bacteria (Leistner and Gould, 2002). As shown in Figure 5, the initial a_*w*_ values of the 5 groups of sausage samples were all around 0.97. With the progress of fermentation, the water was gradually lost and the a_*w*_ value showed a trend of slow decline. At the end of 23rd days of fermentation, the a_*w*_ value dropped to below 0.78 in all groups. From the point of different starter groups at about the 10 days, the a_*w*_ of fermented sausage in LGG, S1, S2, and S4 groups were significantly lower than that in CK group (*p* < 0.05). This may be caused by the fact that lactic acid produced by *L. rhamnosus* at the initial stage of fermentation reduced the pH value of the sausage system, made myosin close to the isoelectric point, changed its binding ability with water, and thus promotes water loss (Hu et al., 2021). At the end of the 23rd day of fermentation, the drying speed of the CK control group may be too fast due to the high pH value, low gelatinization degree of protein and loose texture. While there was a significant difference between the mixed starter culture group and the a_*w*_ of the LGG group, which may be because the yeast mainly grew on the surface of the sausage or the outside of the meat filling during the fermentation process, thus controlling the loss of the moisture activity of the mixed starter culture fermented sausage was significantly higher than that of the LGG group (Ramos-Moreno et al., 2021).

### 3.7. Analysis of POV value of fermented sausage in each group

**FIGURE 6:**
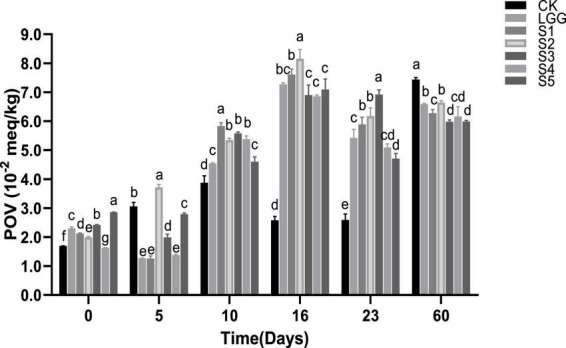
Evolution of peroxide value (POV) value during the ripening of the different samples of sausage. Different letters indicate that POV values of different groups of fermented sausage have significant difference at the same time (*p* < 0.05).

The determination of POV reflects the extent to which sample fat is oxidized to hydroperoxide, and is applicable to the determination of meat product quality at the initial stage of oxidation. As shown in Figure 6, POV value rose at first in the whole process of sausage fermentation and storage. With a trend of decreased slightly at 16−23 days of fermentation, and then increases again at 23−60 days. This is mainly because the hydroperoxide of fat is extremely unstable in the process of lipid oxidation, which is easy to further oxidize to form small molecular compounds such as aldehydes and ketones, thus affecting the accuracy of POV value (Zhang et al., 2021). Kim et al. (2010) also obtained similar results when exploring the influence of different addition forms of garlic and BHA on emulsion-type sausage quality. Between different starter groups, the POV value of fermented sausage in S5 group (with yeast added) was significantly lower than that in LGG group (without yeast added) at 23rd days (*p* < 0.05). In the sausage samples stored for 60 days, the POV value of fermented sausage in S1, S3, S4, and S5 groups was significantly lower than that in CK and LGG groups (*p* < 0.05), indicating that the addition of yeast had a certain inhibitory effect on the change of sausage POV.

### 3.8. Analysis of TBARS value of fermented sausage in each group

Thiobarbituric acid (TBA) is the most commonly used method to evaluate the fat oxidation of meat products. TBARS value represent the contents of all substances that can react with TBA in meat products, mainly the content of malondialdehyde, the secondary product of lipid oxidation, and is a direct indicator of the degree of oxidative deterioration of meat products. Its results are highly related to the sensory quality and the degree of fat oxidation of meat products. Tarladgis et al. (1960) found that the TBARS value was highly correlated with the odor evaluation results of sensory reviewers, with a correlation coefficient of 0.89, and when the TBARS value ranged from 0.5 to 1.0, the reviewers could perceive the rancidity taste.

**FIGURE 7:**
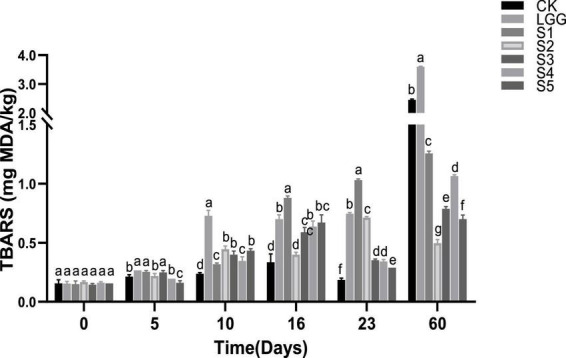
Evolution of thiobarbituric acid reactive substances (TBARS) value during the ripening of the different samples of sausage. Different letters indicate that there are significant differences in TBARS values between different groups at the same time (*p* < 0.05).

As shown in Figure 7, TBARS values show an overall upward trend in the whole process of sausage fermentation and storage. However, TBARS in CK, S3, S4, and S5 groups were slightly reduced on the 23rd day, which may be due to the fact that malondialdehyde reacted with protein in a binding state and was not detected. Except S2 group, the TBARS of sausage in all groups reached the maximum value at 60 days. The TBARS values of S1, S2, S3, S4, and S5 sausage in yeast group were 1.25, 0.50, 0.79, 1.07, and 0.70 mg MDA/kg sausage, respectively. It was significantly lower than that of 2.45 and 3.60 mg MDA/kg sausage in CK and LGG groups (*p* < 0.05), indicating that yeast supplementation could inhibit the increase of TBARS value of sausage.

### 3.9. Analysis of free fatty acid content of fermented sausage in each group

Free fatty acids (FFA) play an important role as precursors to the formation of flavor substances in fermented sausage. The release of a certain amount of FFA during sausage fermentation is conducive to the formation of special flavor of sausage. The effect of different starter on lipid hydrolysis of fermented sausage could be evaluated by measuring the content of FFA in each group. As shown in Table 2, 7 types of saturated fatty acids (SFA), 4 types of monounsaturated fatty acids (MUFA) and 4 types of polyunsaturated fatty acids (PUFA) were detected, respectively in each group of the fermented sausage, and the types of FFA did not change between the groups, which means that microbial fermentation only improved the degree of hydrolysis of FFA, but didn’t change the way of lipid hydrolysis. Chen et al. (2017) also reached a similar conclusion when exploring the effects of bacterial fermentation on lipid decomposition and lipid oxidation in Harbin dry sausage.

**TABLE 2:** Changes of free fatty acids (FFA) content in different fermented sausage samples.

Time (d)	0	23						
FFA (mg/g fat)	Meat mince	CK	LGG	S1	S2	S3	S4	S5
C10:0	0.122 ± 0.006b	0.119 ± 0.006b	0.136 ± 0.006b	0.129 ± 0.009b	0.135 ± 0.005b	0.136 ± 0.013b	0.129 ± 0.004b	0.172 ± 0.004a
C12:0	0.017 ± 0.003cd	0.015 ± 0.008d	0.031 ± 0.005b	0.022 ± 0.003bcd	0.028 ± 0.003bc	0.029 ± 0.004b	0.022 ± 0.002bcd	0.065 ± 0.006a
C14:0	0.498 ± 0.076d	0.538 ± 0.064cd	0.692 ± 0.007b	0.659 ± 0.045b	0.617 ± 0.038bc	0.594 ± 0.049bcd	0.536 ± 0.017cd	1.011 ± 0.069a
C16:0	4.818 ± 0.433d	4.981 ± 0.335cd	6.183 ± 0.062b	5.564 ± 0.226bc	5.276 ± 0.221cd	4.749 ± 0.117d	4.814 ± 0.614d	7.587 ± 0.345a
C17:0	0.098 ± 0.004c	0.086 ± 0.007d	0.136 ± 0.003a	0.121 ± 0.007b	0.095 ± 0.006cd	0.070 ± 0.001e	0.054 ± 0.006f	0.131 ± 0.003ab
C18:0	3.144 ± 0.334b	3.155 ± 0.212b	4.165 ± 0.144a	3.427 ± 0.196b	3.207 ± 0.194b	2.650 ± 0.019c	2.341 ± 0.181c	4.536 ± 0.178a
C20:0	0.098 ± 0.001b	0.096 ± 0.004b	0.117 ± 0.003a	0.097 ± 0.003b	0.096 ± 0.003b	0.088 ± 0.001c	0.080 ± 0.004d	0.102 ± 0.002b
C16:1	0.677 ± 0.046b	0.689 ± 0.071b	0.866 ± 0.016b	0.800 ± 0.051b	0.796 ± 0.045b	0.772 ± 0.050b	0.756 ± 0.089b	1.425 ± 0.229a
C17:1	0.094 ± 0.008c	0.084 ± 0.013cd	0.128 ± 0.001b	0.126 ± 0.006b	0.093 ± 0.007c	0.080 ± 0.002cd	0.071 ± 0.009d	0.162 ± 0.016a
C18:1n9c	7.395 ± 0.811d	7.739 ± 0.572cd	9.840 ± 0.128b	8.730 ± 0.408c	8.046 ± 0.420cd	7.295 ± 0.148d	7.220 ± 0.671d	12.013 ± 0.713a
C20:1	0.250 ± 0.012c	0.264 ± 0.021c	0.360 ± 0.006a	0.310 ± 0.016b	0.293 ± 0.019b	0.249 ± 0.003c	0.212 ± 0.014d	0.298 ± 0.000b
C18:2n6c	5.054 ± 0.584c	4.690 ± 0.415c	5.963 ± 0.062b	5.797 ± 0.374b	5.039 ± 0.309c	4.943 ± 0.187c	4.535 ± 0.156c	7.642 ± 0.476a
C18:3n3	0.306 ± 0.026cd	0.255 ± 0.032d	0.350 ± 0.006bc	0.398 ± 0.038b	0.304 ± 0.021cd	0.343 ± 0.024bc	0.278 ± 0.034cd	0.514 ± 0.068a
C20:2	0.226 ± 0.008cde	0.202 ± 0.018de	0.299 ± 0.012b	0.272 ± 0.029bc	0.248 ± 0.019cd	0.217 ± 0.009de	0.188 ± 0.026e	0.369 ± 0.042a
C20:4n6	0.079 ± 0.004b	0.054 ± 0.006b	0.077 ± 0.016b	0.136 ± 0.018a	0.057 ± 0.007b	0.068 ± 0.004b	0.061 ± 0.003b	0.079 ± 0.026b
SFA	8.795 (38.45%)	8.990 (39.14%)	11.460 (39.06%)	10.019 (37.68%)	9.454 (38.86%)	8.316 (37.32%)	7.976 (37.45%)	13.604 (37.68%)
MUFA	8.416 (36.79%)	8.776 (38.21%)	11.194 (38.15%)	9.966 (37.48%)	9.228 (37.93%)	8.396 (37.68%)	8.259 (38.78%)	13.898 (38.49%)
PUFA	5.665 (24.76%)	5.201 (22.65%)	6.689 (22.8%)	6.603 (24.83%)	5.648 (23.21%)	5.571 (25%)	5.062 (23.77%)	8.604 (23.83%)
Total FFA	22.876	22.967	29.343	26.588	24.330	22.283	21.297	36.106

Values in brackets of SFA, MUFA, and PUFA indicate the proportion of such fatty acids in total free fatty acids (%). Different lower case letters indicate that there is a significant difference in the content of this fatty acid between different groups of fermented sausages (*p* < 0.05).

The CK group is the sausage without inoculating the starters. Compared with the content of fatty acids in the meat mince used for the sausage making, the changes of these two groups are not obvious, with total fatty acids contents of 22.876 mg/g fat and 22.967 mg/g fat, respectively. While total fatty acids contents in LGG, S1, S2, and S5 groups increased to 29.343, 26.588, 24.330, and 36.106 mg/g fat, respectively, indicating that the addition of starter, especially Y12-4, was beneficial to the hydrolytic release of free fatty acids. While for S3 and S4 groups they did not increase the content of free fatty acids in fermented sausages after inoculation, which may be related to the lipase activity of Y12-2 and Y12-3 strains. Another aspect may also be that during the ripening process of sausage fermentation, a large number of unsaturated fatty acids are oxidized to lipid carbonyl compounds, and at the same time, alcohols, aldehydes and ketones are produced, which can form peroxides through free radical chain reaction. These secondary reactions produce a large number of volatile compounds, thereby reducing the content of free fatty acids. According to the proportion of different fatty acids in total fatty acids, the contents of SFA and MUFA were about 38 %, higher than PUFA (22−25 %). Compared with CK group, the proportion of SFA in S1, S2, S3, S4, and S5 groups added with starter decreased, while the proportion of PUFA increased. It is generally believed that the addition of starter is conducive to the hydrolysis of lipids into short-chain volatile fatty acids and other substances, endowing sausages with special flavor, promoting the degradation of SFA and the release of MUFA and PUFA (Hu et al., 2021). According to the types of fatty acids, palmitic acid, stearic acid, oleic acid and linoleic acid were relatively higher in all sausage samples, accounting for about 89% of the total fatty acids, which were the main components of FFA in fermented sausage.

### 3.10. Analysis of volatile flavor substance of fermented sausage in different groups

The determination results of flavor substances in sausage samples are shown in Table 4. A total of 75 volatile flavor substances were analyzed, including 11 aldehydes, 16 alcohols, 10 acids, 9 esters, 4 ketones, 17 terpenes, and 8 other types. The content of terpenes was obviously higher, more than 70% in each sample group, followed by other, acids, alcohols and aldehydes, esters and ketones were relatively less. CK group had the least kinds of flavor compounds (59 substances) in sausages in each group, while LGG and S5 had 67 substances, S1, S3, and S4 had 65 substances, and S2 had 64 substances.

**TABLE 3:** Changes of key volatile flavor compounds OAV in different fermented sausage samples.

	Volatile compounds	Threshold value (μ g/kg)	OVA						
			CK	LGG	S1	S2	S3	S4	S5
1	Hexanal	5.00	7.94 ± 0.21	90.79 ± 2.03	19.53 ± 0.17	5.77 ± 0.76	27.35 ± 1.58	9.42 ± 0.86	30.04 ± 1.77
2	Heptanal	2.80	—	20.83 ± 1.50	—	—	8.95 ± 0.40	—	10.22 ± 0.75
3	Nonanal	1.10	12.63 ± 2.00	55.97 ± 4.31	18.79 ± 0.46	14.04 ± 0.56	18.10 ± 0.99	12.12 ± 1.29	21.29 ± 1.88
4	*(E)*-2-octenal	3.00	—	10.69 ± 1.17	—	—	—	—	—
5	*(E)*-2-nonenal	0.19	—	74.45 ± 4.23	—	—	—	—	—
6	*(E,E)*-2,4-decadienal	0.03	—	122.4 ± 10.98	48.69 ± 4.99	22.50 ± 7.27	40.40 ± 3.71	27.41 ± 2.55	56.61 ± 2.01
7	Hexanol	5.60	0.42 ± 0.04	1.28 ± 0.12	1.41 ± 0.11	0.94 ± 0.03	1.93 ± 0.21	1.27 ± 0.09	2.13 ± 0.09
8	1-octene-3-ol	1.50	2.10 ± 0.17	46.16 ± 4.38	6.20 ± 0.33	3.05 ± 0.30	8.97 ± 0.32	3.93 ± 0.14	12.26 ± 1.22
9	1-heptanol	5.40	0.18 ± 0.04	1.87 ± 0.19	1.07 ± 0.06	0.77 ± 0.11	1.28 ± 0.09	0.94 ± 0.04	1.58 ± 0.19
10	Linalool	0.22	424.05 ± 24.32	389.44 ± 3.04	438.04 ± 12.04	427.18 ± 3.30	374.86 ± 23.42	379.39 ± 2.16	433.17 ± 36.13
11	Ethyl caproate	5.00	1.45 ± 0.12	—	1.77 ± 0.20	1.30 ± 0.12	1.66 ± 0.08	1.58 ± 0.05	2.11 ± 0.08
12	Ethyl octanoate	19.30	0.25 ± 0.02	—	0.40 ± 0.01	0.32 ± 0.02	0.84 ± 0.05	0.38 ± 0.04	1.05 ± 0.04
13	Acetoin	14.00	4.29 ± 0.15	11.19 ± 0.14	6.69 ± 0.30	4.89 ± 0.31	5.82 ± 0.48	4.58 ± 0.42	7.22 ± 0.31
14	α-pinene	14.00	20.88 ± 0.93	17.9 ± 0.05	26.65 ± 2.29	17.14 ± 0.92	14.67 ± 1.36	20.49 ± 1.32	19.17 ± 1.10
15	β-pinene	140.00	2.75 ± 0.14	2.14 ± 0.01	3.00 ± 0.29	2.26 ± 0.10	1.94 ± 0.24	2.39 ± 0.16	2.41 ± 0.14
16	δ-3-carene	770.00	1.88 ± 0.10	1.29 ± 0.06	1.73 ± 0.15	1.47 ± 0.12	1.42 ± 0.24	1.40 ± 0.01	1.78 ± 0.09
17	Sabinene	980.00	0.94 ± 0.07	0.67 ± 0.01	1.16 ± 0.01	0.87 ± 0.07	0.77 ± 0.06	0.98 ± 0.06	0.91 ± 0.06
18	Limonene	200.00	10.45 ± 0.69	9.24 ± 0.17	11.70 ± 1.10	9.39 ± 0.61	8.36 ± 0.46	9.34 ± 0.62	9.90 ± 0.50
19	Styrene	3.60	0.62 ± 0.07	0.65 ± 0.08	1.39 ± 0.10	0.94 ± 0.13	1.47 ± 0.14	1.12 ± 0.02	1.23 ± 0.14
20	β-caryophyllene	64.00	10.61 ± 1.14	9.82 ± 0.70	9.96 ± 0.72	12.97 ± 1.18	9.43 ± 0.49	8.57 ± 0.63	10.23 ± 0.64
21	Allyl methyl sulfide	22.00	4.64 ± 0.22	1.89 ± 0.37	5.39 ± 0.62	5.33 ± 0.45	3.13 ± 0.01	5.16 ± 0.12	1.97 ± 0.07
22	2-pentyl furan	5.80	—	2.95 ± 0.33	—	—	—	—	—
23	*p*-cymene	5.01	96.11 ± 6.52	94.85 ± 1.93	114.70 ± 0.84	92.59 ± 7.47	84.54 ± 5.20	91.64 ± 5.47	95.17 ± 6.01

**TABLE 4:** Changes of volatile flavor substances content in different fermented sausage samples.

	Volatile compounds	Odor	Threshold value (μg/kg)	Probable origin	Sausage samples (μg/kg)							
					CK	LGG	S1	S2	S3	S4	S5	SEM
**Aldehydes**												
1	Hexanal	Grass, tallow, fat	5.00	Lipid oxidation	39.69 ± 1.06	453.95 ± 10.13	97.66 ± 0.84	28.84 ± 3.82	136.73 ± 7.89	47.12 ± 4.28	150.22 ± 8.85	30.24
2	Heptanal	Fat, citrus, rancid	2.80	Lipid oxidation	—	58.31 ± 4.21	—	—	25.05 ± 1.11	—	28.62 ± 2.09	5.24
3	*(E)*-2-hexenal	Apple, green	88.70	Lipid oxidation	—	14.4 ± 1.32	—	—	—	—	—	0.93
4	Nonanal	Fat, citrus, green	1.10	Lipid oxidation	13.89 ± 2.20	61.57 ± 4.75	20.67 ± 0.50	15.45 ± 0.62	19.91 ± 1.09	13.33 ± 1.42	23.42 ± 2.06	3.48
5	*(E)*-2-octenal	Green, nut, fat	3.00	Lipid oxidation	—	32.06 ± 3.5	—	—	—	—	—	2.48
6	Benzaldehyde	Almond, burnt sugar	750.89	Amino acid degradation	—	13.78 ± 0.96	9.31 ± 0.33	12.86 ± 0.94	14.33 ± 0.69	9.56 ± 0.02	13.38 ± 1.30	0.54
7	*(E)*-2-nonenal	Cucumber, fat, green	0.19	Lipid oxidation	—	14.15 ± 0.80	—	—	—	—	—	0.57
8	*(E)*-2-decenal	Tallow	17.00		1.16 ± 0.11	7.58 ± 0.01	1.56 ± 0.57	1.10 ± 0.07	1.58 ± 0.30	0.98 ± 0.22	1.97 ± 0.15	0.45
9	Phenylethanal	Hawthorne, honey, sweet	6.30	Amino acid degradation	—	1.14 ± 0.21	—	—	—	—	—	0.15
10	2-undecenal	Sweet			—	1.94 ± 0.00	—	—	—	—	0.61	0.44
11	*(E,E)*-2,4-decadienal	Fried, wax, fat	0.03		—	3.30 ± 0.30	1.31 ± 0.13	0.61 ± 0.20	1.09 ± 0.10	0.74 ± 0.07	1.53 ± 0.05	0.21
	Total				54.74 ± 3.37	662.18 ± 26.19	130.51 ± 2.37	58.86 ± 5.65	198.69 ± 11.18	71.73 ± 6.01	219.75 ± 14.5	33.00
**Alcohols**												
12	Ethanol	Sweet	950000.00	Carbohydrate fermentation	44.25 ± 5.58	34.6 ± 4.49	94.14 ± 3.44	81.49 ± 4.39	40.51 ± 2.57	54.74	47.96 ± 1.88	5.57
13	2-heptanol	Mushroom	65.24	Lipid β oxidation	0.25 ± 0.10	2.07 ± 0.17	3.37 ± 0.19	2.87 ± 0.06	2.28 ± 0.19	2.34 ± 0.20	1.92 ± 0.13	0.20
14	Hexanol	Resin, flower, green	5.60	Lipid oxidation	2.34 ± 0.24	7.17 ± 0.68	7.92 ± 0.62	5.26 ± 0.19	10.81 ± 1.17	7.12 ± 0.53	11.95 ± 0.49	0.79
15	2-octanol	Mushroom, fat	7.80	Lipid oxidation		0.24 ± 0.05	0.19 ± 0.06	0.17 ± 0.01	0.30 ± 0.05	0.18 ± 0.08	0.17 ± 0.00	0.02
16	1-octene-3-ol	Mushroom	1.50	Lipid β oxidation	3.16 ± 0.25	69.25 ± 6.57	9.31 ± 0.49	4.57 ± 0.45	13.46 ± 0.49	5.89 ± 0.22	18.39 ± 1.83	4.73
17	1-heptanol	Chemical, green	5.40	Lipid oxidation	0.98 ± 0.24	10.11 ± 1.02	5.77 ± 0.34	4.17 ± 0.58	6.92 ± 0.47	5.08 ± 0.19	8.52 ± 1.00	0.69
18	2-ethyl-1-hexanol	Rose, green	25482.20	Lipid oxidation	49.41 ± 2.84	40.46 ± 1.31	55.49 ± 2.00	35.10 ± 1.88	31.13 ± 3.40	36.08 ± 1.86	52.28 ± 4.18	1.97
19	*(E)*-2-hepten-1-ol	Pungent, fatty, plastic	4172.00		—	—	—	—	0.67 ± 0.02	0.41 ± 0.03	0.79 ± 0.10	0.06
20	2-nonanol	Cucumber	58.00		—	0.83 ± 0.07	1.4 ± 0.11	1.09 ± 0.02	0.97 ± 0.13	0.96 ± 0.02	0.43 ± 0.01	0.08
21	2,3-butanediol	Fruit, onion	20000.00	Carbohydrate fermentation	51.65 ± 2.86	—	44.55 ± 1.77	21.59 ± 2.07	46.58 ± 5.05	5.77 ± 1.12	6.24 ± 0.08	5.26
22	Linalool	Flower, lavender	0.22	Spices	93.29 ± 5.35	85.68 ± 0.67	96.37 ± 2.65	93.98 ± 0.73	82.47 ± 5.15	83.47 ± 0.47	95.3 ± 7.95	1.58
23	Octanol	Chemical, metal, burnt	125.80	Lipid oxidation	2.77 ± 0.28	8.76 ± 0.86	4.07 ± 0.25	3.47 ± 0.24	4.66 ± 0.16	2.17 ± 0.16	4.36 ± 0.53	0.42
24	α-terpineol	Oil, anise, mint	1200.00	Spices	11.61 ± 1.93	11.99 ± 0.66	11.18 ± 0.73	10.30 ± 0.62	11.10 ± 1.64	11.77 ± 0.50	13.25 ± 0.74	0.32
25	Myrtenol	Sweet, mint, medical	7.00		0.48 ± 0.05	0.55 ± 0.11	0.62 ± 0.01	0.39 ± 0.03	0.43 ± 0.03	0.73 ± 0.11	0.55 ± 0.05	0.03
26	Benzylalcohol	Sweet, flower	2546.21	Amino acid degradation	0.87 ± 0.07	1.22 ± 0.16	1.83 ± 0.15	1.82 ± 0.11	1.85 ± 0.20	1.46 ± 0.10	2.31 ± 0.16	0.11
27	Phenethylalcohol	Honey, spice, rose, lilac	564.23	Smoking	5.31 ± 0.52	6.53 ± 0.51	13.62 ± 0.61	13.16 ± 0.64	13.36 ± 0.75	11.91 ± 0.61	19.48 ± 1.51	1.04
	Total				266.37 ± 20.31	279.46 ± 17.33	349.83 ± 13.42	279.43 ± 12.02	267.5 ± 21.47	230.08 ± 6.2	283.9 ± 20.64	14.76
**Acids**												
28	Acetic acid	Sour	99000.00	Carbohydrate fermentation	43.17 ± 2.81	191.18 ± 6.10	242.04 ± 2.63	213.12 ± 1.80	247.98 ± 50.83	156.09 ± 14.11	196.84 ± 10.58	15.56
29	Propionic acid	Pungent, rancid, soy	2190.00	Lipid oxidation	—	—	3.32 ± 0.23	3.44 ± 0.34	3.66 ± 0.31	1.28 ± 0.26	2.46 ± 0.23	0.23
30	Butyric acid	Rancid, cheese, sweat	2400.00	Lipid oxidation	13.92 ± 0.47	37.35 ± 2.36	54.05 ± 3.21	53.6 ± 2.26	51.15 ± 6.76	40.39 ± 3.39	58.99 ± 5.75	3.33
31	Isovaleric acid	Sweat, acid, rancid	490.00	Amino acid degradation	5.55 ± 0.55	6.18 ± 0.58	15.33 ± 0.53	8.18 ± 0.36	11.44 ± 1.24	8.22 ± 1.74	10.87 ± 0.61	0.78
32	Pentanoic acid	Sweat	11000.00	Lipid oxidation	0.44 ± 0.06	2.51 ± 0.04	1.61 ± 0.10	2.41 ± 0.24	4.2 ± 0.51	2.44 ± 0.18	5.29 ± 0.58	0.40
33	Caproic acid	Sweat	890.00	Lipid oxidation	1.9 ± 0.21	19.18 ± 2.88	12.02 ± 0.54	12 ± 1.15	15.27 ± 1.32	11.43 ± 0.86	22.11 ± 0.75	1.47
34	Heptanoic acid	Rancid, sour, cheesy, sweat	640.00	Lipid oxidation	—	2.66 ± 0.11	2.73 ± 0.21	2.88 ± 0.13	3.94 ± 0.44	3.05 ± 0.15	5.29 ± 0.57	0.26
35	Octanoic acid	Sweat, cheese	3000.00	Lipid oxidation	0.89 ± 0.02	6.81 ± 0.32	5.88 ± 0.54	5.68 ± 0.29	9.44 ± 0.92	7.09 ± 0.01	15.99 ± 2.64	1.19
36	Nonanoic acid	Green, fat	4600.00	Lipid oxidation	0.49 ± 0.01	1.74 ± 0.17	2.22 ± 0.15	2.97 ± 0.21	4.03 ± 0.33	3.09 ± 0.69	10.49 ± 2.95	0.89
37	Decanoic acid	Rancid, fat	10000.00	Lipid oxidation	—	2.4 ± 0.03	2.85 ± 0.24	3.18 ± 0.24	5.24 ± 0.42	3.08 ± 0.37	8.13 ± 2.88	0.74
	Total				66.36 ± 4.13	270.01 ± 12.59	342.05 ± 8.38	307.46 ± 7.02	356.35 ± 63.08	236.16 ± 21.76	336.46 ± 27.54	24.57
**Esters**												
38	Methyl hexanoate	Fruit, fresh, sweet	70.00		11.91 ± 2.93	—	22.89 ± 0.40	11.20 ± 0.82	—	18.31 ± 1.76	—	1.60
39	Ethyl caproate	Apple peel, fruit	5.00	Esterase activity	7.25 ± 0.61	—	8.84 ± 1.00	6.48 ± 0.59	8.32 ± 0.38	7.88 ± 0.25	10.57 ± 0.41	0.32
40	Ethyl lactate	Fruit	50000.00		1.17 ± 0.18	3.67 ± 0.03	4.68 ± 0.12	6.01 ± 0.80	5.12 ± 0.41	5.10 ± 0.34	6.17 ± 0.47	0.45
41	Methyl octanoate	Orange	200.00	Esterase activity	—	4.75 ± 0.62	4.85 ± 0.00	2.60 ± 0.15	4.23 ± 0.10	4.01 ± 0.29	4.21 ± 0.08	0.20
42	Butyl hexanoate	Fruit	10000.00		0.71 ± 0.11	—	—	—	—	—	—	0.06
43	Ethyl octanoate	Fruit	19.30	Esterase activity	4.81 ± 0.44	—	7.75 ± 0.24	6.27 ± 0.29	16.22 ± 1.00	7.33 ± 0.71	20.25 ± 0.78	1.53
44	Ethyl nonanoate	Fruity, rose, waxy, wine	377.00		0.93	1.45 ± 0.03	0.82 ± 0.56	—	0.43 ± 0.06	0.63 ± 0.01	1.70 ± 0.03	0.16
45	γ-hexalactone	Sweet, coumarin	260.00	Lipid oxidation	0.19	0.36 ± 0.03	0.32 ± 0.03	0.27 ± 0.06	—	—	0.30 ± 0.10	0.02
46	γ-nonalactone	Coconut, peach	9.70		0.37 ± 0.06	1.05 ± 0.02	1.10 ± 0.10	1.24 ± 0.04	1.73 ± 0.08	1.07 ± 0.06	1.88 ± 0.12	0.11
	Total				27.34 ± 4.33	11.28 ± 0.73	51.25 ± 2.45	34.07 ± 2.75	36.05 ± 2.03	44.33 ± 3.42	45.08 ± 1.99	3.23
**Ketones**												
47	2-nonanone	Hot milk, soap, green	41.00	Lipid β oxidation	5.59 ± 0.43	—	—	—	—	—	—	0.22
48	Piperitone	Mint, fresh	680.00		1.23 ± 0.05	1.19 ± 0.08	1.32 ± 0.04	1.35 ± 0.08	1.27 ± 0.15	1.47 ± 0.09	1.38 ± 0.09	0.03
49	DL-carvone	Mint, basil, fennel	27.00		0.56 ± 0.01	0.83 ± 0.08	0.48 ± 0.09	0.42 ± 0.01	0.84 ± 0.08	0.53 ± 0.02	0.58 ± 0.06	0.04
50	Acetoin	Butter, cream	14.00	Carbohydrate fermentation	60.13 ± 2.09	156.72 ± 1.91	93.63 ± 4.21	68.51 ± 4.29	81.45 ± 6.74	64.07 ± 5.92	101.08 ± 4.36	6.67
	Total				67.51 ± 2.58	158.74 ± 2.07	95.43 ± 4.34	70.28 ± 4.38	83.56 ± 6.97	66.07 ± 6.03	103.04 ± 4.51	9.00
**Terpenes**												
51	α-pinene	Pine, turpentine	14.00	Spices	292.27 ± 13.04	250.63 ± 0.75	373.08 ± 32	240.02 ± 12.88	205.37 ± 19.04	286.85 ± 18.43	268.35 ± 15.44	11.40
52	β-pinene	Pine, resin, turpentine	140.00	Spices	384.37 ± 20.03	299.76 ± 1.68	419.58 ± 40.01	315.93 ± 14.46	271.52 ± 34.04	334 ± 22.51	337.72 ± 19.01	11.22
53	δ-3-carene	Lemon, resin	770.00		1445.16 ± 79.51	993.24 ± 49.07	1329.65 ± 117.93	1130.71 ± 88.87	1092.93 ± 184.61	1079.57 ± 4.63	1373.1 ± 71.94	39.59
54	Sabinene	Pepper, turpentine, wood	980.00	Spices	925.03 ± 65.23	658.18 ± 9.21	1139.96 ± 7.93	848.04 ± 65.04	751.56 ± 58.69	964.74 ± 60.42	892.36 ± 63.16	31.06
55	α-terpinene	Lemon	80.00	Spices	36.80 ± 4.12	31.00 ± 1.61	59.13 ± 5.72	47.33 ± 3.14	40.74 ± 3.63	45.64 ± 3.17	47.55 ± 3.40	2.07
56	Limonene	Lemon, orange	200.00	Spices	2090.01 ± 137.5	1847.49 ± 33.96	2340.64 ± 219.39	1878.23 ± 122.99	1671.00 ± 91.50	1868.14 ± 124.24	1979.72 ± 100.26	50.03
57	β-phellandrene	Mint, turpentine	500.00	Spices	91.63 ± 8.75	65.48 ± 2.15	112.04 ± 12.16	81.27 ± 6.19	71.57 ± 4.77	87.37 ± 5.03	88.53 ± 5.42	3.14
58	γ-terpinene	Gasoline, turpentine	1000.00	Spices	33.9 ± 4.62	33.28 ± 2.23	40.47 ± 1.40	34.21 ± 2.78	23.63 ± 0.19	35.68 ± 2.37	27.10 ± 2.56	1.22
59	(Z)-β-ocimene	Citrus, herb, flower	34.00	Spices	9.38 ± 1.14	6.02 ± 0.04	10.05 ± 0.08	8.86 ± 0.60	9.59 ± 1.08	9.52 ± 0.52	8.01 ± 0.73	0.32
60	Styrene	Balsamic, gasoline	3.60	Spices	2.25 ± 0.24	2.33 ± 0.30	5.00 ± 0.35	3.38 ± 0.48	5.30 ± 0.51	4.02 ± 0.07	4.44 ± 0.49	0.29
61	Terpinolene	Pine, plastic	200.00	Spices	95.88 ± 12.55	68.06 ± 3.19	117.31 ± 9.46	96.93 ± 8.65	79.59 ± 3.28	98.84 ± 9.77	98.18 ± 6.24	3.33
62	α-p-dimethylstyrene	Citrus, pine	85.00		26.69 ± 0.96	25.42 ± 2.53	31.68 ± 1.66	29.91 ± 0.29	26.25 ± 3.52	27.11 ± 0.57	30.43 ± 1.31	0.63
63	δ-elemene	Wood		Spices	48.16 ± 5.11	46.63 ± 3.65	50.88 ± 4.36	57.66 ± 5.37	65.55 ± 13.05	48.40 ± 2.21	54.95 ± 2.89	1.99
64	β-caryophyllene	Wood, spice	64.00	Spices	679.23 ± 72.74	628.61 ± 44.65	637.20 ± 45.95	829.81 ± 75.50	603.47 ± 31.34	548.22 ± 40.17	654.56 ± 41.18	19.99
65	α-humulene	Wood	160.00	Spices	36.2 ± 5.9	32.8 ± 2.94	32.61 ± 1.78	45.83 ± 5.44	32.58 ± 2.51	27.14 ± 0.48	35.95 ± 2.49	1.34
66	δ-cadinene	Thyme, medicine, wood		Spices	6.18 ± 0.18	6.02 ± 0.50	5.66 ± 1.18	7.10 ± 0.38	4.88 ± 0.31	4.45 ± 0.01	6.33 ± 0.60	0.22
67	Caryophyllene oxide	Herb, sweet, spice	410.00		1.71 ± 0.23	2.63 ± 0.19	1.49 ± 0.12	1.21 ± 0.10	1.14 ± 0.15	0.74 ± 0.01	1.28 ± 0.13	0.12
	Total				6204.85 ± 431.85	4997.58 ± 158.65	6706.43 ± 501.48	5656.43 ± 413.16	4956.67 ± 452.22	5470.43 ± 294.61	5908.56 ± 337.25	303.49
**Others**												
68	Allyl methyl sulfide	Garlic, onion	22.00		102.04 ± 4.94	41.61 ± 8.03	118.55 ± 13.71	117.26 ± 9.88	68.82 ± 0.14	113.56 ± 2.54	43.39 ± 1.57	7.67
69	2-pentyl furan	Green bean, butter	5.80	Lipid oxidation	—	17.10 ± 1.94	—	—	—	—	—	1.37
70	p-cymene	Solvent, gasoline, citrus	5.01		481.51 ± 32.66	475.18 ± 9.67	574.63 ± 4.21	463.88 ± 37.43	423.53 ± 26.04	459.12 ± 27.42	476.82 ± 30.10	10.22
71	D-camphor	Camphor, minty, phenolic, herbal	1360.00		5.22 ± 0.16	5.1 ± 0.79	5.78 ± 0.50	4.66 ± 0.23	5.08 ± 0.28	5.62 ± 0.16	5.59 ± 0.50	0.11
72	Borneol	Camphor	180.00		0.26 ± 0.07	0.33 ± 0.06	0.33 ± 0.05	0.52 ± 0.05	0.49 ± 0.05	0.41 ± 0.04	0.47 ± 0.03	0.02
73	Phenol	Phenol	58585.25	Amino acid degradation	0.88 ± 0.07	0.91 ± 0.01	0.9 ± 0.12	0.84 ± 0.01	0.96 ± 0.11	0.75 ± 0.05	0.82 ± 0.03	0.02
74	p-cresol	Medicine, phenol, smoke	3.90		1.82 ± 0.07	1.66 ± 0.05	1.99 ± 0.11	2.16 ± 0.07	2.16 ± 0.17	1.89 ± 0.07	2.64 ± 0.32	0.07
75	Meta-cresol	Fecal, plastic	15.00		4.87 ± 0.11	4.64 ± 0.45	5.26 ± 0.35	5.11 ± 0.10	4.81 ± 0.41	4.43 ± 0.15	6.01 ± 0.54	0.12
	Total				596.6 ± 38.08	546.53 ± 21	707.44 ± 19.05	594.43 ± 47.77	505.85 ± 27.2	585.78 ± 30.43	535.74 ± 33.09	41.99

SEM is the Standard Error of Mean (SEM) of the same volatile compound content in different groups of sausage samples.

Aldehydes mainly come from the automatic oxidation of unsaturated fatty acids such as oleic acid and linoleic acid and the degradation of amino acids. They have low threshold value. Therefore, although the total content of aldehydes is low, they are of great significance to the formation of sausage flavor. The total content of aldehydes in LGG group was the highest (662.18 ± 26.19 μg/kg), which was much higher than S2 group (58.86 ± 5.65 μg/kg) and S4 group (71.73 ± 6.01 μg/kg). Hexanal, as the aldehyde with the highest proportion, was considered to be positively correlated with the degree of fat oxidation (Olivares et al., 2011). The content of hexaldehyde in S1−S5 groups was 97.66 ± 0.84, 28.84 ± 3.82, 136.73 ± 7.89, 47.12 ± 4.28, and 150.22 ± 8.85 μg/kg, which was much lower than 453.95 ± 10.13 μg/kg in LGG group. The results showed that LGG group had the highest lipid oxidation degree, while yeast fermentation effectively inhibited the production of hexanal, which had good antioxidant effect, consistent with the results of POV value and TBARS. In addition, heptanal, nonanal, (*E*)-2-decenal also found the same change. (*E*)-2-hexenal, (*E*)-2-octenal, (*E*)-2-nonenal and phenylacetaldehyde only detected in LGG group.

The OAV value of flavor compounds can directly reflect the contribution of compounds to the formation of sausage flavor. It is generally believed that flavor compounds with OAV ≥ 1 are the key flavor compounds of the product, and the higher the OAV value, the stronger the contribution. Table 3 collated the key flavor compounds in the 23 days sausage sample. It isn’t difficult to find that the OVA value of aldehydes was high, which plays an important role in the flavor formation of sausage. Among them, hexanal, heptanal, nonanal, (*E*)-2-octenal, (*E*)-2-nonenal, (*E,E*)-2, 4-decadienal are the key flavor aldehydes, which have grass, rancid, fat, nut, cucumber and fried flavor, respectively.

The formation of alcohols is mainly related to microbial carbohydrate fermentation, such as ethanol and 2, 3-butanediol. However, it is difficult to have a significant impact on the overall flavor of sausage because of their high threshold. Some alcohols are associated with fat β-oxidation and auto-oxidation, such as 1-octene-3-ol (with mushroom flavor), which has higher content in LGG group. On the whole, the content of alcohols in S1 group was higher, which may be related to the strong carbohydrate capacity of Y3-1. Among the key flavor alcohols, hexanol, 1-octene-3-ol and 1-heptanol are mainly derived from lipid oxidation and β-oxidation, and have resin, mushroom and pharmaceutical flavor, respectively, while linalool is mainly derived from spice and has flower flavor.

The contents of acids, especially acetic acid and butyric acid, in the sausage samples inoculated with starter were much higher than those in the CK group. Acetic acid is mainly produced by the metabolism of carbohydrates by microorganisms, and butyric acid is thought to be related to the automatic oxidation of fat. Similar to alcohols, acids also had higher thresholds, and no key flavor acids were found in OVA analysis.

The production of esters is mainly affected by microbial esterase activity, and most of them are aromatic. Esters generated by short-chain fatty acids mostly have fruit flavor, while esters generated by long-chain fatty acids mostly have fat flavor. It is easily to find from Table 4 that, the addition of yeast facilitates the formation of esters. The content of esters in S1 group was the highest (51.25 ± 2.45 μg/kg), which may be related to the high content of acids and alcohols in S1 group, and then promoted the formation of esters. Cano-García et al. (2014) also reached a similar conclusion when exploring the influence of *D. hansenii* on aroma substances of fermented sausage. Among the key flavor esters, ethyl caproate and ethyl octanoate had fruit flavor, which were detected in all groups except LGG.

Ketones may be derived from lipid oxidation or raw meat. Four ketones were detected in 7 groups of sausage samples, of which 2-nonanone (related to lipid β oxidation) was only detected in CK group. 3-hydroxy-2-butanone (acetoin) was detected in all groups, OVA > 1, and the highest content in LGG group. It was associated with microbial carbohydrate fermentation, and had a pleasant buttery odor and was thought to contribute significantly to the formation of sausage flavor.

Alkenes accounted for the highest proportion of the flavor substances, mainly from the use of spices, and α-pinene (pine), β-pinene (pine resin), δ-3-carene (lemon), sabinene (pepper), limonene (lemon), and β-caryophyllene (wood) were relatively high. In addition, a certain amount of allyl methyl sulfide, 2-pentyl furan, *p*-cymene and *D*-camphor were also detected in various sausage samples. 2-pentyl furan is related to the automatic oxidation of fat and is a secondary oxidation product of linoleic acid, which exists in most preserved meat products (Shahidi and Wanasundara, 1998). *p*-cymene had a citrus flavor and had little difference in content among different groups, which might be derived from the use of spices in sausage making process.

### 3.11. Analysis of sensory evaluation of fermented sausage in each group

**FIGURE 8:**
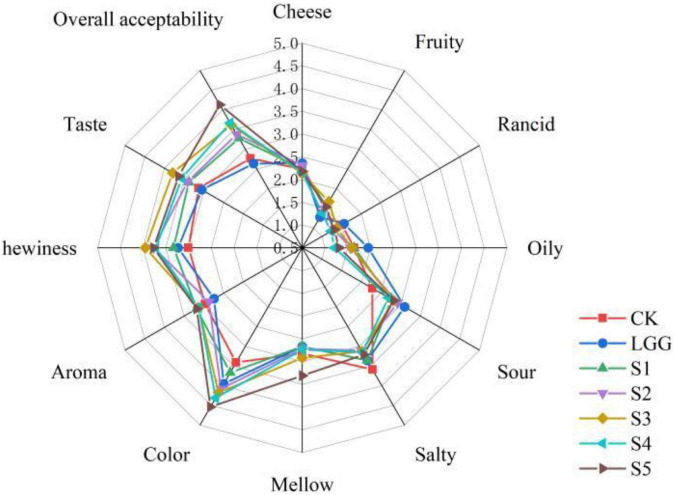
Radar image of sensory evaluation scores of different fermented sausage samples.

Although many literatures have indicated that moderate fat oxidation can improve the flavor and quality of fermented meat products, excessive oxidation will produce rancidity and rancid taste, shorten the shelf life, and ultimately affect the acceptance of products. However, there is no clear definition of the highest sensory acceptance of products at what level of fat oxidation, and fermented sausage is a relatively complex system. Therefore, it is difficult to explore the effect of different yeast on the actual sensory quality of fermented sausage by analyzing the degree of lipid oxidation and the content of flavor substances. The sensory evaluation results of 7 groups of fermented sausages by 39 professionally trained food students are shown in Figure 8.

The higher cheese flavor in the LGG group may be partly due to higher levels of 3-hydroxy-2-butanone (cheese, butter flavor). The fruit flavor in the yeast groups were significantly higher than that of LGG group and CK group, and the rancid taste and oily taste were decreased. The score of S4 group was the lowest (1.23 ± 1.22 and 1.21 ± 1.15), and that of LGG group was the highest (1.56 ± 1.59 and 1.95 ± 1.73), which were consistent with the previous content of aldehydes in volatile flavor substances, indicating that the use of yeast improved the production of flavor substances related to lipid oxidation. In terms of sour taste, the LGG group scored the highest, while the yeast group scored lower, which may be related to the yeast’s ability to utilize and metabolize organic acids. In terms of odor (comprehensive score), taste (comprehensive score) and mellow taste, compared with LGG group, scores of yeast groups had increased except S1 group. It is generally believed that yeast plays an important role in the formation of flavor substances such as short peptides and esters during the maturation of fermented meat products due to its ability to decompose protein and fat.

In terms of overall taste, the overall taste of S4 and S5 was higher, reaching 3.67 ± 1.20 and 4.13 ± 1.28, while the overall taste of LGG group and CK group was lower, partly due to the stimulating sour taste of LGG group and the single taste of CK group. S5 group has good performance in mellow taste, appearance color, odor, texture and taste, indicating that this group is the most popular. In addition, S4 group performed well in appearance color, smell and taste, and there was no significant difference in the content of volatile flavor aldehydes between group S4 and S2 (the lowest group). Therefore, the S4 and S5 groups of sausage samples were selected to analyze the flavor substances and metagenomic related indicators of sausage samples after storage.

### 3.12. Analysis of volatile flavor compounds in fermented sausage after storage

Based on above fat oxidation, volatile flavor compounds analysis and sensory evaluation results, two groups of sausage samples S4 and S5 with low fat oxidation level and excellent sensory scores were screened out. At the same time, the volatile flavor substances of the sausage samples of LGG group stored until 60 days and 0 day of CK were measured. The results were compared based on the 23rd day results, as shown in Table 5. A total of 78 volatile flavor compounds were identified in stored sausage samples, including 16 aldehydes, 16 alcohols, 10 acids, 8 esters, 3 ketones, 17 terpenes, and 8 others.

**TABLE 5:** Changes of volatile flavor compounds in fermented sausage samples after storage.

	Volatile compounds	Odor	Threshold value (μg/kg)	Probable origin	0 day	23 days			60 days			
					CK	LGG	S4	S5	LGG	S4	S5	SEM
**Aldehydes**												
1	Hexanal	Grass, tallow, fat	5.00	Lipid oxidation	—	453.95 ± 10.13	47.12 ± 4.28	150.22 ± 8.85	540.59 ± 4.72	272.83 ± 22.84	227.12 ± 10.42	41.31
2	Heptanal	Fat, citrus, rancid	2.80	Lipid oxidation	—	58.31 ± 4.21	—	28.62 ± 2.09	74.74 ± 1.61	41.23 ± 3.24	39.13 ± 2.10	4.47
3	*(E)*-2-hexenal	Apple, green	88.70	Lipid oxidation	—	14.40 ± 1.32	—	—	12.91 ± 1.24	5.86 ± 0.73	5.68 ± 0.45	1.32
4	Octanal	Fat, soap, lemon, green	0.59	Lipid oxidation	—	—	—	—	95.94 ± 22.21	51.64 ± 2.74	51.78 ± 3.90	7.56
5	(Z)-2-heptenal		56.00	Lipid oxidation	—	—	—	—	143.37 ± 13.14	56.53 ± 4.72	56.08 ± 5.21	13.49
6	Nonanal	Fat, citrus, green	1.10	Lipid oxidation	1.56 ± 0.31	61.57 ± 4.75	13.33 ± 1.42	23.42 ± 2.06	76.76 ± 0.31	41.36 ± 2.59	43.89 ± 2.56	4.98
7	*(E)*-2-octenal	Green, nut, fat	3.00	Lipid oxidation	—	32.06 ± 3.5	—	—	56.66 ± 5.10	—	—	6.29
8	*(E,E)*-2,4-heptadienal	Fatty, green, cinnamon	57.00		—	—	—	—	2.13 ± 0.19	—	—	0.13
9	Benzaldehyde	Almond, burnt sugar	750.89	Amino acid degradation	4.05 ± 0.61	13.78 ± 0.96	9.56 ± 0.02	13.38 ± 1.30	17.83 ± 0.41	15.83 ± 1.44	17.41 ± 1.64	0.71
10	*(E)*-2-nonenal	Cucumber, fat, green	0.19	Lipid oxidation	—	14.15 ± 0.8	—	—	24.94 ± 1.95	—	—	2.72
11	*(E)*-2-decenal	Tallow	17.00			7.58 ± 0.01	0.98 ± 0.22	1.97 ± 0.15	12.3 ± 1.16	5.54 ± 0.45	5.33 ± 0.34	0.94
12	Phenylethanal	Hawthorne, honey, sweet	6.30	Amino acid degradation		1.14 ± 0.21	—	—	—	—	—	0.15
13	Ethyl benzaldehyde	Sweet	13.00						3.63 ± 0.26			0.18
14	2-undecenal	Sweet			—	1.94 ± 0.00	—	0.61	5.32 ± 0.49	2.29 ± 0.09	2.55 ± 0.21	0.42
15	*(E,E)*-2,4-decadienal	Fried, wax, fat	0.03		—	3.30 ± 0.30	0.74 ± 0.07	1.53 ± 0.05	13.44 ± 2.21	4.84 ± 0.69	4.07 ± 0.61	1.16
16	Hexadecanal	Cardboard			—	—	—	—	1.73 ± 0.11	4.24 ± 0.79	4.49 ± 0.28	0.47
	Total				5.61 ± 0.92	662.18 ± 26.19	71.73 ± 6.01	219.75 ± 14.5	1082.29 ± 55.11	502.19 ± 40.32	457.53 ± 27.72	69.92
**Alcohols**												
17	Ethanol	Sweet	950000.00	Carbohydrate fermentation	—	34.6 ± 4.49	54.74	47.96 ± 1.88	33.26 ± 7.42	33.89 ± 6.61	26.30 ± 9.73	2.90
18	2-heptanol	Mushroom	65.24	Lipid β oxidation	—	2.07 ± 0.17	2.34 ± 0.20	1.92 ± 0.13	1.03 ± 0.15	1.95 ± 0.21	1.54 ± 0.17	0.11
19	Hexanol	Resin, flower, green	5.60	Lipid oxidation	0.78 ± 0.13	7.17 ± 0.68	7.12 ± 0.53	11.95 ± 0.49	13.26 ± 1.22	39.10 ± 2.92	38.83 ± 1.19	3.66
20	2-octanol	Mushroom, fat	7.80	Lipid oxidation	—	0.24 ± 0.05	0.18 ± 0.08	0.17 ± 0.00	—	—	—	0.02
21	1-octene-3-ol	Mushroom	1.50	Lipid β oxidation	0.98 ± 0.08	69.25 ± 6.57	5.89 ± 0.22	18.39 ± 1.83	120.99 ± 7.96	54.81 ± 3.63	55.62 ± 4.92	9.11
22	1-heptanol	Chemical, green	5.40	Lipid oxidation	—	10.11 ± 1.02	5.08 ± 0.19	8.52 ± 1.00	13.19 ± 1.04	16.92 ± 2.08	20.42 ± 0.61	1.21
23	2-ethyl-1-hexanol	Rose, green	25482.20	Lipid oxidation	30.28 ± 1.1	40.46 ± 1.31	36.08 ± 1.86	52.28 ± 4.18	20.56 ± 2.81	20.64 ± 1.38	27.52 ± 1.91	2.78
24	*(E)*-2-hepten-1-ol	Pungent, fatty, plastic	4172.00		—	—	0.41 ± 0.03	0.79 ± 0.1	—	—	—	0.10
25	2-nonanol	Cucumber	58.00		—	0.83 ± 0.07	0.96 ± 0.02	0.43 ± 0.01	—	—	—	0.10
26	2,3-butanediol	Fruit, onion	20000.00	Carbohydrate fermentation	—	—	5.77 ± 1.12	6.24 ± 0.08	—	39.44 ± 4.05	35.08 ± 2.00	5.31
27	Linalool	Flower, lavender	0.22	Spices	57.80 ± 1.47	85.68 ± 0.67	83.47 ± 0.47	95.30 ± 7.95	86.55 ± 5.95	96.56 ± 5.33	105.72 ± 4.64	2.06
28	Octanol	Chemical, metal, burnt	125.80	Lipid oxidation	0.33 ± 0.17	8.76 ± 0.86	2.17 ± 0.16	4.36 ± 0.53	9.33 ± 0.84	5.98 ± 0.69	6.04 ± 0.04	0.61
29	α-terpineol	Oil, anise, mint	1200.00	Spices	7.82 ± 0.71	11.99 ± 0.66	11.77 ± 0.50	13.25 ± 0.74	11.75 ± 1.11	13.43 ± 0.67	13.23 ± 2.88	0.36
30	Myrtenol	Sweet, mint, medical	7.00		—	0.55 ± 0.11	0.73 ± 0.11	0.55 ± 0.05	0.65 ± 0.08	0.65 ± 0.12	—	0.03
31	Benzylalcohol	Sweet, flower	2546.21	Amino acid degradation	0.48 ± 0.05	1.22 ± 0.16	1.46 ± 0.10	2.31 ± 0.16	1.31 ± 0.31	2.06 ± 0.10	2.2 ± 0.02	0.11
32	Phenethylalcohol	Honey, spice, rose	564.23	Smoking	0.31 ± 0.01	6.53 ± 0.51	11.91 ± 0.61	19.48 ± 1.51	3.88 ± 0.65	8.32 ± 0.35	8.50 ± 1.19	1.15
	Total				98.78 ± 3.72	279.46 ± 17.33	230.08 ± 6.20	283.90 ± 20.64	315.76 ± 29.54	333.75 ± 28.14	341.00 ± 29.3	15.24
**Acids**												
33	Acetic acid	Sour	99000.00	Carbohydrate fermentation	—	191.18 ± 6.10	156.09 ± 14.11	196.84 ± 10.58	185.09 ± 0.57	187.10 ± 17.7	194.98 ± 18.58	4.46
34	Propionic acid	Pungent, rancid, soy	2190.00	Lipid oxidation	—	—	1.28 ± 0.26	2.46 ± 0.23	4.03 ± 0.30	3.45 ± 0.28	2.95 ± 0.40	0.25
35	Butyric acid	Rancid, cheese, sweat	2400.00	Lipid oxidation	1.71 ± 0.81	37.35 ± 2.36	40.39 ± 3.39	58.99 ± 5.75	47.97 ± 2.96	63.52 ± 2.88	63.96 ± 2.46	2.72
36	Isovaleric acid	Sweat, acid, rancid	490.00	Amino acid degradation	—	6.18 ± 0.58	8.22 ± 1.74	10.87 ± 0.61	—	—	—	0.82
37	Pentanoic acid	Sweat	11000.00	Lipid oxidation	—	2.51 ± 0.04	2.44 ± 0.18	5.29 ± 0.58	9.65 ± 0.42	8.42 ± 0.29	8.39 ± 0.15	0.76
38	Caproic acid	Sweat	890.00	Lipid oxidation	0.87 ± 0.3	19.18 ± 2.88	11.43 ± 0.86	22.11 ± 0.75	88.12 ± 3.21	64.46 ± 2.84	70.46 ± 1.76	7.32
39	Heptanoic acid	Rancid, sour, cheesy, sweat	640.00	Lipid oxidation	—	2.66 ± 0.11	3.05 ± 0.15	5.29 ± 0.57	6.98 ± 0.52	8.62 ± 0.6	8.23 ± 0.62	0.59
40	Octanoic acid	Sweat, cheese	3000.00	Lipid oxidation	0.31 ± 0.01	6.81 ± 0.32	7.09 ± 0.01	15.99 ± 2.64	24.55 ± 1.32	31.44 ± 2.31	32.24 ± 2.94	2.70
41	Nonanoic acid	Green, fat	4600.00	Lipid oxidation	1.61 ± 0.18	1.74 ± 0.17	3.09 ± 0.69	10.49 ± 2.95	11.18 ± 3.15	15.49 ± 1.45	12.94 ± 1.82	1.32
42	Decanoic acid	Rancid, fat	10000.00	Lipid oxidation	—	2.4 ± 0.03	3.08 ± 0.37	8.13 ± 2.88	13.49 ± 1.00	21.56 ± 2.90	24.14 ± 1.58	2.15
	Total				4.50 ± 1.30	270.01 ± 12.59	236.16 ± 21.76	336.46 ± 27.54	391.06 ± 13.45	404.06 ± 31.25	418.29 ± 30.31	25.28
**Esters**												
43	Methyl hexanoate	Fruit, fresh, sweet	70.00		0.89 ± 0.5	—	18.31 ± 1.76	—	—	—	—	1.02
44	Ethyl caproate	Apple peel, fruit	5.00	Esterase activity	—	—	7.88 ± 0.25	10.57 ± 0.41	—	—	—	0.61
45	Ethyl lactate	Fruit	50000.00		—	3.67 ± 0.03	5.10 ± 0.34	6.17 ± 0.47	3.44 ± 0.17	5.10 ± 0.50	3.71 ± 0.32	0.27
46	Methyl octanoate	Orange	200.00	Esterase activity	1.57 ± 0.10	4.75 ± 0.62	4.01 ± 0.29	4.21 ± 0.08	4.11 ± 0.35	4.17 ± 0.35	4.76 ± 0.34	0.10
47	Ethyl octanoate	Fruit	19.30	Esterase activity	—	—	7.33 ± 0.71	20.25 ± 0.78	—	33.86 ± 1.42	34.01 ± 1.50	3.07
48	Ethyl nonanoate	Fruity, rose, waxy, wine	377.00		—	1.45 ± 0.03	0.63 ± 0.01	1.7 ± 0.03	1.83 ± 0.28	1.29 ± 0.93	—	0.18
49	γ-hexalactone	Sweet, coumarin	260.00	Lipid oxidation	—	0.36 ± 0.03	—	0.3 ± 0.1	1.5 ± 0.13	1.71 ± 0.08	1.29 ± 0.16	0.17
50	γ-nonalactone	Coconut, peach	9.70		—	1.05 ± 0.02	1.07 ± 0.06	1.88 ± 0.12	1.61 ± 0.03	3.54 ± 0.36	3.35 ± 0.15	0.26
	Total				2.46 ± 0.6	11.28 ± 0.73	44.33 ± 3.42	45.08 ± 1.99	12.49 ± 0.96	49.67 ± 3.64	47.12 ± 2.47	4.27
**Ketones**												
51	Piperitone	Mint, fresh	680.00		0.68 ± 0.07	1.19 ± 0.08	1.47 ± 0.09	1.38 ± 0.09	1.52 ± 0.07	1.66 ± 0.07	1.71 ± 0.16	0.05
52	DL-carvone	Mint, basil, fennel	27.00		0.33 ± 0.02	0.83 ± 0.08	0.53 ± 0.02	0.58 ± 0.06	1.28 ± 0.04	1.09 ± 0.13	0.76 ± 0.10	0.06
53	Acetoin	Butter, cream	14.00	Carbohydrate fermentation	6.42 ± 0.38	156.72 ± 1.91	64.07 ± 5.92	101.08 ± 4.36	—	—	—	13.64
	Total				7.43 ± 0.47	158.74 ± 2.07	66.07 ± 6.03	103.04 ± 4.51	2.80 ± 0.11	2.75 ± 0.20	2.47 ± 0.26	12.46
**Terpenes**												
54	α-pinene	Pine, turpentine	14.00	Spices	73.64 ± 6.59	250.63 ± 0.75	286.85 ± 18.43	268.35 ± 15.44	328.58 ± 53.06	344.37 ± 15.44	374.00 ± 24.60	11.35
55	β-pinene	Pine, resin, turpentine	140.00	Spices	112.30 ± 9.09	299.76 ± 1.68	334.00 ± 22.51	337.72 ± 19.01	375.35 ± 16.7	409.00 ± 16.06	407.52 ± 23.75	10.23
56	δ-3-carene	Lemon, resin	770.00		558.34 ± 50.50	993.24 ± 49.07	1079.57 ± 4.63	1373.1 ± 71.94	1197.6 ± 23.69	1567.96 ± 190.84	1692.08 ± 75.63	62.96
57	Sabinene	Pepper, turpentine, wood	980.00	Spices	51.78 ± 16.14	658.18 ± 9.21	964.74 ± 60.42	892.36 ± 63.16	743.99 ± 115.18	1025.37 ± 27.42	1050.91 ± 34.82	34.14
58	α-terpinene	Lemon	80.00	Spices	11.32 ± 1.24	31.00 ± 1.61	45.64 ± 3.17	47.55 ± 3.40	29.42 ± 2.81	49.52 ± 2.09	59.25 ± 0.13	2.63
59	Limonene	Lemon, orange	200.00	Spices	668.46 ± 48.21	1847.49 ± 33.96	1868.14 ± 124.24	1979.72 ± 100.26	2093.74 ± 174.12	2294.97 ± 83.77	2303.58 ± 150.27	48.58
60	β-phellandrene	Mint, turpentine	500.00	Spices	25.55 ± 1.42	65.48 ± 2.15	87.37 ± 5.03	88.53 ± 5.42	74.35 ± 4.2	91.78 ± 7.78	114.07 ± 8.67	3.80
61	γ-terpinene	Gasoline, turpentine	1000.00	Spices	8.11 ± 0.58	33.28 ± 2.23	35.68 ± 2.37	27.1 ± 2.56	38.41 ± 1.42	39.74 ± 4.96	47.05 ± 4.20	1.70
62	(Z)-β-ocimene	Citrus, herb, flower	34.00	Spices	1.50 ± 0.50	6.02 ± 0.04	9.52 ± 0.52	8.01 ± 0.73	4.08 ± 0.79	7.15 ± 1.48	10.16 ± 0.06	0.48
63	Styrene	Balsamic, gasoline	3.60	Spices	1.01 ± 0.15	2.33 ± 0.30	4.02 ± 0.07	4.44 ± 0.49	3 ± 0.47	5.53 ± 0.24	4.77 ± 0.27	0.28
64	Terpinolene	Pine, plastic	200.00	Spices	35.85 ± 1.90	68.06 ± 3.19	98.84 ± 9.77	98.18 ± 6.24	81.63 ± 11.73	110.50 ± 5.38	120.17 ± 4.81	4.08
65	α-p-dimethylstyrene	Citrus, pine	85.00		14.71 ± 1.03	25.42 ± 2.53	27.11 ± 0.57	30.43 ± 1.31	28.88 ± 1.44	30.31 ± 1.41	30.63 ± 3.86	0.67
66	δ-elemene	Wood		Spices	15.00 ± 2.93	46.63 ± 3.65	48.40 ± 2.21	54.95 ± 2.89	54.86 ± 4.74	60.35 ± 5.25	68.53 ± 3.19	1.90
67	β-caryophyllene	Wood, spice	64.00	Spices	286.46 ± 57.41	628.61 ± 44.65	548.22 ± 40.17	654.56 ± 41.18	759.46 ± 44.52	755.29 ± 63.33	802.57 ± 41.99	22.39
68	α-humulene	Wood	160.00	Spices	16.64 ± 2.97	32.8 ± 2.94	27.14 ± 0.48	35.95 ± 2.49	39.52 ± 3.85	40.25 ± 3.73	45.1 ± 1.67	1.39
69	δ-cadinene	Thyme, medicine, wood		Spices	3.08 ± 0.16	6.02 ± 0.50	4.45 ± 0.01	6.33 ± 0.60	6.60 ± 0.39	7.51 ± 0.42	7.50 ± 0.53	0.27
70	Caryophyllene oxide	Herb, sweet, spice	410.00		0.36 ± 0.06	2.63 ± 0.19	0.74 ± 0.01	1.28 ± 0.13	3.16 ± 0.27	1.91 ± 0.48	2.20 ± 0.22	0.19
	Total				1884.11 ± 200.88	4997.58 ± 158.65	5470.43 ± 294.61	5908.56 ± 337.25	5862.63 ± 459.38	6841.51 ± 430.08	7140.09 ± 378.67	273.69
**Others**												
71	Allyl methyl sulfide	Garlic, onion	22.00		72.10 ± 4.10	41.61 ± 8.03	113.56 ± 2.54	43.39 ± 1.57	22.50 ± 13.85	53.63 ± 3.42	27.77 ± 17.35	8.56
72	2-pentyl furan	Green bean, butter	5.80	Lipid oxidation	—	17.10 ± 1.94	—	—	58.18 ± 2.30	40.17 ± 2.16	42.55 ± 3.18	4.01
73	p-cymene	Solvent, gasoline, citrus	5.01		183.45 ± 23.56	475.18 ± 9.67	459.12 ± 27.42	476.82 ± 30.10	533.19 ± 27.55	556.53 ± 13.34	573.53 ± 35.92	11.53
74	D-camphor	Camphor, minty, phenolic, herbal	1360.00		2.30 ± 0.53	5.10 ± 0.79	5.62 ± 0.16	5.59 ± 0.5	8.50 ± 0.28	7.18 ± 0.32	7.12 ± 0.38	0.28
75	Borneol	Camphor	180.00		0.22 ± 0.08	0.33 ± 0.06	0.41 ± 0.04	0.47 ± 0.03	0.41 ± 0.03	0.38 ± 0.03	0.34 ± 0.11	0.02
76	Phenol	Phenol	58585.25	Amino acid degradation	0.41 ± 0.04	0.91 ± 0.01	0.75 ± 0.05	0.82 ± 0.03	0.24 ± 0.13	1.13 ± 0.15	1.05 ± 0.07	0.07
77	p-cresol	Medicine, phenol, smoke	3.90		1.55 ± 0.11	1.66 ± 0.05	1.89 ± 0.07	2.64 ± 0.32	1.52 ± 0.14	2.85 ± 0.19	2.79 ± 0.32	0.14
78	Meta-cresol	Fecal, plastic	15.00		4.26 ± 0.24	4.64 ± 0.45	4.43 ± 0.15	6.01 ± 0.54	5.12 ± 0.27	6.56 ± 0.45	6.85 ± 0.57	0.25
	Total				264.29 ± 28.66	546.53 ± 21.00	585.78 ± 30.43	535.74 ± 33.09	629.66 ± 44.55	668.43 ± 20.06	662.00 ± 57.90	30.49

SEM is the Standard Error of Mean (SEM) of the same volatile compound content in different groups of sausage samples.

Compared with the results on day 23, 5 aldehydes were increased, which were octanal, (*Z*)-2-heptenal, (*E,E*)-2, 4-heptadienal, ethyl benzaldehyde and hexadecanal, which were mainly related to the automatic oxidation of fat. Moreover, the proportion of aldehydes in total volatile components increased significantly, indicating that the oxidation degree of fat in sausage increased with the increase of storage time, which was consistent with the previous results of POV and TBARS values of sausage samples after storage. Compared with fresh sausage, the types and proportions of other volatile compounds in stored sausage didn’t change significantly. According to the sausage sample groups, 43 flavor compounds were detected in the CK- 0 d group, with the least species. 67, 65, and 67 flavor compounds were detected in LGG-23d, S4-23d, and S5-23d, respectively, and 68, 66 and 64 flavor compounds were detected in LGG-60d, S4-60d, and S5-60d, respectively.

**FIGURE 9:**
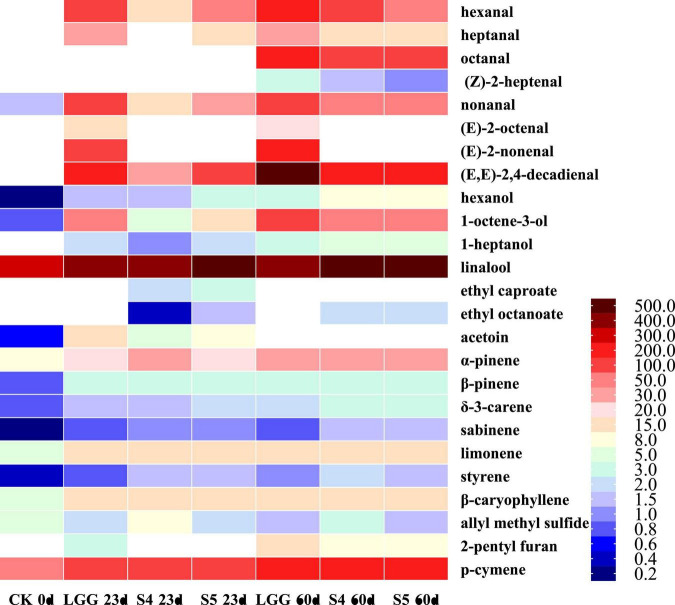
OAV heat map of key volatile flavor compounds in different fermented sausage samples after storage.

Compared with the fresh samples at day 23, the content of aldehydes in the stored samples at day 60 was significantly increased, and the total content of aldehydes in LGG-60d group was 1082.29 ± 55.11 μg/kg, significantly higher than LGG-23d (662.18 ± 26.19 μg/kg), S4-60d (502.19 ± 40.32 μg/kg), and S5-60d (457.53 ± 27.72 μg/kg). It should be noted that the aldehydes content of the S4 group with lower aldehydes content at day 23 exceeded that of the S5 group at day 60. The contents of almost all aldehydes increased after storage, and the content of LGG-60 d group was much higher than that of yeast group, among which the contents of hexanal, octanal, nonanal, (*E*)-2-octenal, (*E*)-2-decenal, (*E,E*)-2, 4-decadienal were significantly different. According to the OAV thermal map analysis (Figure 9), it was not difficult to find that the OAV of aldehydes changed significantly (blue indicated lower OAV value, while red indicated higher OAV value), indicating that aldehydes had an important contribution to the formation of unpleasant odor. Octanal and (*Z*)-2-heptenal were the newly added key flavor aldehydes.

The contents of alcohols in samples increased slightly after storage, and the contents of group S4-60d were the highest, reached up to 333.75 ± 28.14 μg/kg. The contents of ethanol and 2-ethyl-1-hexanol decreased after storage, while the contents of hexanol, 1-octene-3-ol, 1-heptanol and 2, 3-butanediol increased after storage. The concentration of 1-octene-3-ol increased by 9.3 times and 3.0 times in S4-60d and S5-60d, respectively. 1-octene-3-ol has a mushroom flavor at low concentration, but is considered to be the main component of peculiar smell at high concentration (Zhou et al., 2020). The variation of linalool content may be related to the water loss of sausages after storage, which was verified by the variation of a_*w*_.

The content of acids in samples was increased after storage, some acid substances had obvious changes, such as butyric acid, pentanoic acid, caproic acid, heptanoic acid, octanoic acid, nonanoic acid and decanoic acid, but still did not reach the odor threshold, so further discussion is not required. The total content of esters did not change significantly in the samples after storage, among which methyl caproate and ethyl caproate were difficult to be detected after storage, while that of ethyl caproate increased. Wu et al. (2019) found that the content of esters decreased at first and then increased during the storage of air-dried sausage, which may be related to the formation of esters through Maillard reaction during storage.

Acetoin, the highest content of ketones, was only detected in fresh and mature samples, but not detected after storage, while the contents of other ketones did not change significantly. The content of terpenes in all sample groups was increased, mainly from the use of spices. The main reason for the increase in content is related to the water loss in the storage process of sausage. Among the other compounds, the content of 2-pentyl furan (related to lipid oxidation) increased in all groups, similar to the change of aldehydes, the content of 2-pentyl furan in LG-60d group was the highest, reaching 58.18 ± 2.3 μg/kg.

### 3.13. Exploration of functional genes related to lipid oxidation in yeast

**FIGURE 10:**
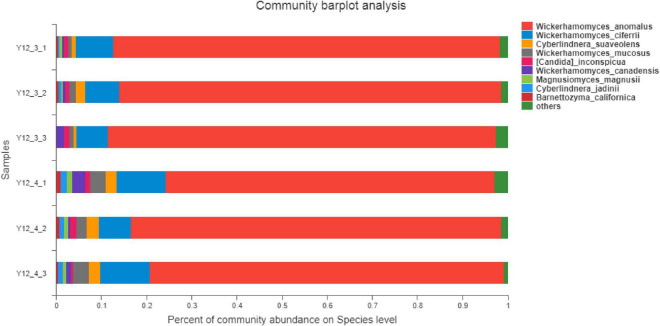
Community abundance at species level of fungi in fermented sausage.

**FIGURE 11:**
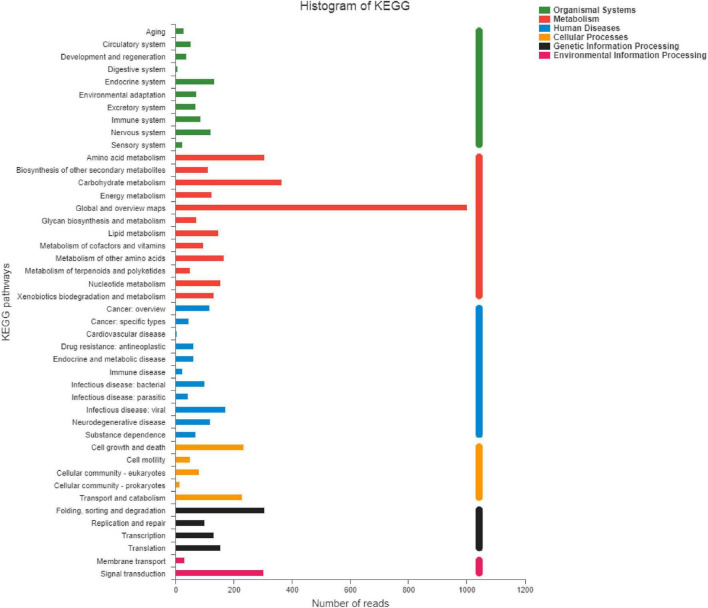
Pathway classification statistics of *Wickerhamomyces anomalus* Y12-3.

Based on the results of previous determination of volatile flavor compounds in salami, combined with OAV values and possible production pathways of flavor compounds, hexanal, heptanal, nonanal, (*E*)-2-decenal, (*E,E*)-2, 4-decdienal and other flavor compounds related to fat oxidation were selected as the flavor compounds to focus on. The fungal community composition of the samples can be used to analyze the community abundance of fermented sausage at the level of fungal species. The relative abundance of 9 fungi was greater than 0.01 % in the colony structure of fermented sausage, as shown in Figure 10. Among them, *Wickerhamomyces anomalus*, *Wickerhamomyces ciferrii*, *Cyberlindnera suaveolens*, and *Wickerhamomyces mucosus* accounted for about 95 %. The proportion of *W. anomalus* in group Y12-3 was higher than that in Y12-4, and the repeatability was better. Therefore, the subsequent analysis of functional genes in Y12-3 group could reduce the influence of fungi such as *W. ciferrii* on characteristic flavor substances to a certain extent. The *W. anomalus* Y12-3 gene set was constructed, which was conducted KEGG function annotation and Pathway classification statistics, and the results were shown in Figure 11.

The Level 2 functional genes belonging to the same Level 1 were represented in the same color, with the functional gene names for different levels 2 on the vertical axis and the abundance values for the functional gene on the horizontal axis. The abundance of genes related to Metabolism was significantly higher in all six metabolic pathways. Secondary KEGG annotation was performed on genes related to metabolic pathways, of which global and overview maps abundance were the highest, followed by Carbohydrate metabolism, Amino acid metabolism, Metabolism of other amino acids, Nucleotide metabolism, and Lipid metabolism. Meanwhile, the existence of Aldehyde Dehydrogenase (ALDH) function gene could be annotated in the Y12-3 gene set through KEGG database comparison, which was related to Aldehyde metabolism.

Aldehyde Dehydrogenase refers to a class of aldehyde dehydrogenases that rely on NAD(P)+ to catalyze the oxidation of aldehydes. The reaction pathway of its catalytic oxidation of aldehydes is shown in Figure 11. Aldehydes, NAD(P)^+^ and H_2_O undergo redox reactions under the catalysis of ALDH to produce carboxylate, NAD(P)H and H^+^. Xu et al. (2020) analyzed the effects of 8 *W. anomalus* strains on lipid oxidation of 4°C frozen surimi and found that the addition of yeasts J3, J7, J8, J9, J12, and J18 could significantly reduce the content of TBARS in surimi (*p* < 0.05), and the scavenging rate was proportional to the activity of ALDH. Ziegler and Vasiliou (1999) summarized the research status of ALDH super family and indicated that ALDH super family members can catalyze exogenous and endogenous aldehydes in a broad spectrum. ALDH1A1, ALDH1A2, ALDH1A3, ALDH3B1, and ALDH8A1 can effectively reduce aldehydes produced by lipid peroxidation such as 4-hydroxynonenal, propanal, hexanal, octanal, n-decanal, benzaldehyde, and malondialdehyde.

**FIGURE 12:**
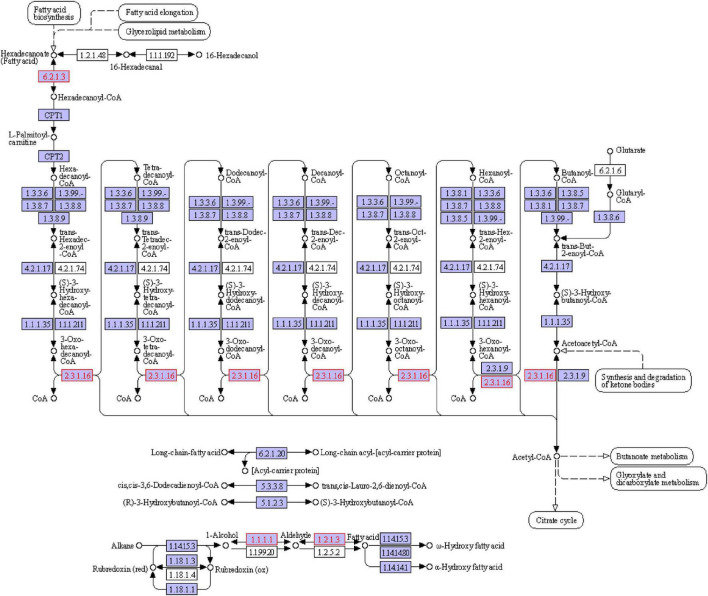
Diagram of fatty acid degradation metabolic pathway.

**FIGURE 13:**
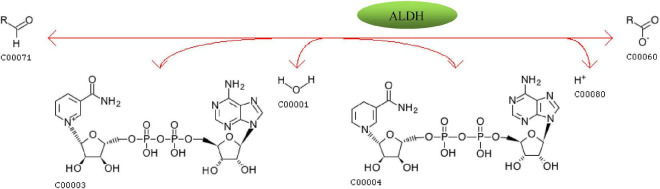
The oxidation equation of aldehydes catalyzed by aldehyde dehydrogenase.

Aldehyde Dehydrogenase plays the function of metabolic aldehydes through Fatty acid degradation (Pathway ID: ko00071), and the related genes identified include aldehyde dehydrogenase (ppa:PAS_chr3_0987) and long-chain acyl-CoA synthase (kmx:KLMA_70312), as shown in Figure 12 and Table 6. Alcohols form their corresponding aldehydes under the action of alcohol dehydrogenase (EC: 1.1.1.1), and the aldehydes can produce corresponding fatty acids under the action of ALDH. Meanwhile, it should be noted that the above two processes are reversible reactions. According to the results of volatile flavor substances in 23 days sausage samples, except for caproic acid and valeric acid, the content of acids related to lipid oxidation in S4 group was higher than that in LGG group, which verified the possibility that Y12-3 could reduce the content of aldehydes in sausage by metabolizing aldehydes through ALDH (Figure 13).

**TABLE 6:** Key genes and enzymes involved in the degradation of fatty acid in *Wickerhamomyces anomalus* Y12-3.

KEGG gene	KO	KEGG pathway	KEGG enzyme	Enzyme
ppa:PAS_c hr3_0987	K00128	ko00010, ko00053, ko00071, ko00280, ko00310, ko00330, ko00340, ko00380, ko00410, ko00561, ko00620, ko00625, ko00903, ko00981	1.2.1.3	Aldehyde dehydrogenases
kmx:KLM A_70312	K01897	ko00061, ko00071, ko02024, ko03320, ko04146, ko 04216, ko04714, ko04920	6.2.1.3	Long chain acyl-coA synthase

## 4. Conclusion

The antioxidant capacity of five yeast strains isolated from fermented food was evaluated *in vitro* based on H_2_O_2_ tolerance, DPPH radical, superoxide anion radical, hydroxyl radical scavenging activity of each cell component (complete bacterial suspension, supernatant after crushing), all these five strains showed the potential of antioxidant activity. However, more attention should be paid to the antioxidation of starter in sausage and whether yeast still retains strong antioxidation after long storage. Considering if yeast is used alone for sausage production without the acid producing and bacteriostatic effects of lactic acid bacteria, it will lead to quality and safety problems in the sausage. *Lactobacillus rhamnosus* YL-1 is a strain screened in our laboratory with strong acid production, nitrite degradation and bacteriostatic effect, and it was mixed with yeast together to apply to fermented salami sausage. At the same time, it could be used alone as the experimental control (LGG group) to obtain more accurate results. Compared with LGG group, the addition of yeast effectively reduced the POV value and TBARS value of sausage, especially in Y12-3 and Y12-4 groups. The analysis results of volatile flavor substances showed that the aldehyde content of LGG group added with lactic acid bacteria alone was significantly higher than that of other groups. After the co fermentation with yeast, the aldehyde content was significantly reduced, especially Y12-3 and Y4-1 groups. The content of flavor substances related to lipid oxidation in Y12-3 group, such as hexanal (fat flavor), heptaldehyde (rancid flavor), *(E)*-2-nonenal (yellow melon flavor), *(E, E)*-2,4-decadienal (fried flavor), was significantly reduced. This result was also supported by sensory evaluation. In Y12-3 group, the scores of putrid taste and oily taste were significantly lower, and the overall acceptance was good, and the oxidation of fat was still effectively inhibited at 60 days. Species and functional contribution analysis showed that yeast Y12-3 contributed the most to the overall function of fungi, and its genes involved in the metabolic pathway were the most abundant at KEGG level1, mainly involved in the global and overview map, carbohydrate metabolism and amino acid metabolism. Two genes related to fatty acid degradation of aldehydes (ko00071) pathway were found by Y12-3 gene mining. As the key enzyme of aldehyde metabolism, ALDH can direct catalytic aldehyde oxidative dehydrogenation to generate acid substances, which can be used as the follow-up and enzyme activity and gene regulation object, further clarify its role in aldehyde metabolism.

Yeast can be used as a potential antioxidant starter in the production of fermented sausage. The research and development of sausage has practical application value by exploring the antioxidant effect of the starter itself. In the above research, although the metagenomics technology was used to analyze the composition of fungal colonies and predict the gene function, the metabolic pathway of yeast to the lipid oxidation related flavor substances in salami sausage was obtained, and the related enzymes were speculated, the activity of aldehyde dehydrogenase in yeast still needs further verification and reasonable regulation.

## Data availability statement

The original contributions presented in this study are included in the article/supplementary material, further inquiries can be directed to the corresponding author.

## Author contributions

YL designed and drafted the manuscript. YC carried out the physicochemical properties test and sausage making. KY performed the analysis of microbial populations part and provided helpful feedback and revised the manuscript. SZ performed analysis of volatile flavor compounds. QY helped in antioxidant activities of yeast strains measurement *in vitro*. JW assisted in securing funding and managed the project. All authors contributed to the article and approved the submitted version.
